# Review of subtribe Singilina Jeannel, 1949, of the Middle East and Central Asia (Coleoptera, Carabidae, Lebiini)

**DOI:** 10.3897/zookeys.155.1779

**Published:** 2011-12-15

**Authors:** Alexander Anichtchenko

**Affiliations:** 1 Daugavpils University, Institute of Systematic Biology, Vienibas iela 13-229, Daugavpils LV-5400, Latvia

**Keywords:** Coleoptera, Carabidae, *Singilis*, new species, redescription, country records, review, taxonomy

## Abstract

Species of the genus *Singilis* Rambur, 1837 (*Phloeozeteus* Peyron, 1856, **syn. n.**, *Agatus* Motschulsky, 1845, **syn. n.**), occurring in the Middle East and Central Asia are reviewed, with 24 species now recognized in the region, including ten species described as new: *Singilis makarovi* **sp. n.** (Tajikistan), *Singilis jedlickai* **sp. n.** (Afghanistan), *Singilis kolesnichenkoi* **sp. n.** (Iran), *Singilis kabakovi* **sp. n.** (Afghanistan, Iran), *Singilis timuri* **sp. n.** (Uzbekistan), *Singilis klimenkoi* **sp. n.** (Iran), *Singilis saeedi* **sp. n.** (Iran), *Singilis felixi* **sp. n.** (UAE), *Singilis kryzhanovskii* **sp. n.** (Iran, Turkmenistan), and *Singilis timidus* **sp. n.** (Iran); *Singilis libani* (Sahlberg, 1913) is recognized as a valid species; and *Singilis solskyi* **nom. n.** is proposed as a replacement name for *Agatus bicolor* (Solsky, 1874, not Rambur 1837), now placed in *Singilis* as junior homonym. New synonymies include: *Singilis cingulatus* (Gebler, 1843) = *Singilis jakeschi* Jedlička, 1967, **syn. n.;** *Singilis mesopotamicus* Pic, 1901 = *Singilis apicalis* Jedlička, 1956, **syn. n.** A key to species is provided. Habitus and aedeagal illustrations are provided for all species. Distributional data include many new country records.

## Introduction

This study aims to clarify the circumscription of the subtribe Singilina Jeannel, 1949, and the status of its member taxa. Until now, most species of this group have been poorly defined, rarely collected, and hard to identify.

The most recent catalogue covering the study area (Lorenz 2005) lists two species of *Singilis* Rambur, 1837, eight species of *Phloeozeteus* Peyron, 1856, and seven species of *Agatus* Motschulsky, 1845. This study leads us to conclude that the fauna of the region includes 24 species, all in the genus *Singilis* Rambur (=*Phloeozeteus* Peyron, *Agatus* Motschulsky).

## Material and methods

This study was based on a total of about 270 specimens, including a number of primary types. Measurements: body length, from anterior margin of clypeus to apex of elytra along suture; length of pronotum, along midline; width of pronotum, at widest point; length of elytra, from his base to apex along suture; and width of elytra, at widest point.

The material from the following institutional and private collections has been examined:

AAC	Alexander Anichtchenko private collection (Spain)

APC	Andreas Pütz private collection (Germany)

DUBC	Daugavpils University Beetle Collection (Latvia)

DWWC	Dawid W. Wrase collection (Germany)

IZEC	Institute of Zoology Collection (Armenia)

KOC	K. Orszulik private collection (Czech Republic)

MMBC	Moravske Muzeum, Brno (Czech Republic)

MNHN	National Museum of Natural History in Paris (France)

MPU	Collection of Zoology & Ecology Department, Moscow State Pedagogical University (Russia)

NHMW	Naturhistorisches Museum Wien (Austria)

NMPC	National Museum of Natural History, Prague (Czech Republic)

RFC	Ron Felix private collection (The Netherlands)

RMNH	Natural History Museum ‘Naturalis', Leiden (The Netherlands)

SMNS	Staatliches Museum für Naturkunde Stuttgart (Germany)

UAEIC	United Arab Emirates Invertebrate Collection (UAE)

ZIN	Zoological Institute, St. Petersburg (Russia)

ZMM	Zoological Museum of Moscow University (Russia)

ZSM	Zoologische Staatssammlung München (Germany)

High-resolution habitus images of *Singilis* species, including type specimens and additional material, are available at http://www.carabidae.pro.

## Subtribe Singilina Jeannel, 1949

Jeannel (1949: 915) proposed the tribe Singilini to include *Singilis* Rambur, 1837; *Phloeozeteus* Peyron, 1856; *Paralebia* Peringuey, 1898; *Somotrichus* Seidlitz, 1887; *Pephrica* Alluaud, 1936; *Paulianites* Jeannel, 1949; and *Velindopsis* Burgeon, 1937; genera whose adults are characterized by small size, and pale, hairy integument. Mateu (1963) excluded from the tribe all the genera but *Singilis*, with *Phloeozeteus* as a subgenus. Ball and Hilchie (1983: 197) treated Singilini as a junior synonym of Dromiina.

The exact composition of this subtribe has not been settled. Debate on the taxonomic status of *Singilis* and *Phloeozeteus* began almost immediately after their description (Reiche 1860; Schaum 1860) and continued until the recent work of Mateu (1963). The taxonomic status of another related genus, *Agatus* Motschulsky, 1845, treated until now in the subtribe Agrina Kirby, 1837 (Kabak 2003; Lorenz 2005), has never been reconsidered since Motschulsky proposed the genus. Motschulsky (1845) considered it to be close to *Calleida* Latreille, 1824, based on similarities in the maxillary palpi and pectinate tarsal claws, although both features are widespread among Lebiini of several subtribes. *Agatus*, *Phloeozeteus*, and *Singilis* were defined so poorly that Jedlička (1956, 1961a, 1961b, 1963a, 1963b) at various points repeatedly moved species between those ‘genera'. *Agatus irakensis* (Jedlička 1963a: 5) was recently synonymized with *Lebia (Singili* sstr.) *syriaca* Pic, 1901 (Anichtchenko 2011). Based on our study of more extensive material, we propose the synonymy *Agatus* Motschulsky, 1845 syn. n. = *Phloeozeteus* Peyron, 1856 syn. n. =*Singilis* Rambur, 1837.

The recent catalogues (Kabak 2003; Lorenz 2005) treat the subtribe Singilina as including two genera, *Singilis* and *Phloeozeteus*, with 62 recognized species. The vast majority is found in Africa and the Middle East; a few are known to occur in the south Mediterranean, Central Asia, and India.

### Genus Singilis

Rambur, 1837

http://species-id.net/wiki/Singilis

Singilis Rambur 1837: 25 Type species: *Singilis bicolor* Rambur, 1837 (nec Solsky 1874)
Agatus Motschulsky 1845: 10. Type species *Glycia fasciata* Motschulsky, 1844 (= *Dromius cingulatus* Gebler, 1843: 37) syn. n.
Phloeozeteus Peyron 1856: 715. Type species *Coptodera plagiata* Reiche & Saulcy, 1855 syn. n.
Paralebia Peringuey 1898: 335. Type species *Paralebia vicaria* Peringuey, 1898
Phloezetaeus : Jedlička 1956: 204 [unavailable]
Phloeozetaeus : Jedlička 1961a: 3 [unavailable]
Phloeozetoeus : Jedlička 1961b: 163 [unavailable]
Phloeozetteus : Jedlička 1963a: 6 [unavailable]
Phloeozetus : Kabak 2003: 438 [unavailable]

#### Remarks.

Now we are not in position to give diagnostic features of this genus, for we do not know the other related genera well enough. The limits of these generic groups are not yet defined. Details of relationships among genera and subtribes remain to be worked out. In lieu of a definitive treatment of classification of *Singilis*, we adopt here the position of Ball and Hilchie (1983). Now a revision of this genus is in progress. In this, the author will try to clarify its position among closely related genera, and will discuss some characters and methods proposed by Basilewsky (1984) and Casale (1998). Inferring phylogenetic relationships within Lebiini from characters of the female reproductive tract will be offered.

The genus *Singilis* as treated here may not be monophyletic; however, the sheer numbers and morphological diversity of its species, both described and undescribed, make a comprehensive revision of the group unfeasible at this time. Still, we believe that describing distinctive species will help faunal studies and contribute to the understanding of higher taxonomy.

#### Singilis flavipes

(Solsky, 1874)

http://species-id.net/wiki/Singilis_flavipes

Glycia flavipes Solsky 1874: 35
Agatus afghanus Jedlička 1961b: 163

##### Material examined.

AFGHANISTAN: ♂ – TYPE, red label: Agatus afganus sp. n. det. Ing. Jedlička; white label: Afganistan, Bhougavi; Cotype ♂, Afghanistan, Douchi; Cotype ♀, Afghanistan, Dehran (NMPC). KAZAKHSTAN: S Kazakhstan, Karatau Mt. Rng., 40 km N Igilik vill., Kurkal, N43°47'003", E68°03'138", 543m, 8.V.2010, light trap, Ivanov A.V. leg. (5♂♂ 3♀♀, AAC). TURKMENISTAN: Amudar'a riv., Chardzhou, 15.V.1993, Kamarkovskii A. leg. (1♂, AAC); Kaahkinskij raj., 28.IV–25.V.1994, leg. A. Kalninsh (1♂, DUBC).

##### Diagnosis.

This species is most similar to the sympatric *Singilis cingulatus*. The two species can be diagnosed easily by different color of legs, i.e., *Singilis cingulatus* has femora and apical part of tibiae piceous to black (Figs 2–3).

**Figures 1–5.**
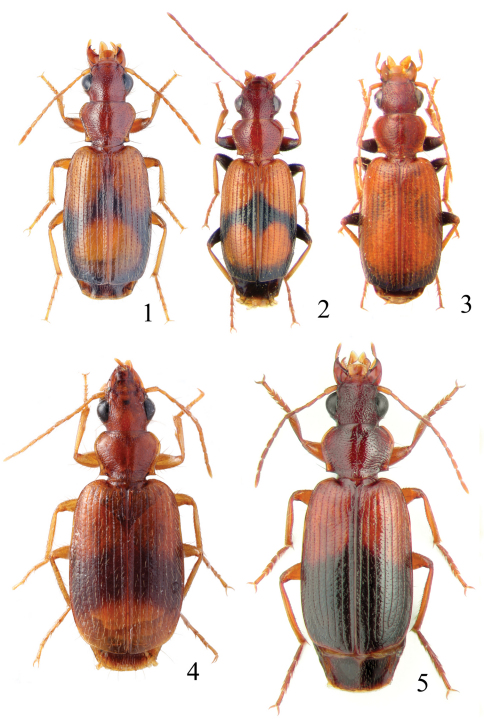
Habitus of *Singilis*: **1** *Singilis flavipes* (Kazakhstan) **2–3** *Singilis cingulatus* (Kazakhstan) **4** *Singilis amoenulus* (SW Turkmenistan, Beki-Bent) **5** *Singilis solskyi* nom.n. (Kazakhstan).

##### Redescription.

Length 4.3–5.3 mm. Body elongate; head and pronotum red-brown, elytra black with red-brown basal band and a common preapical round red macula reaching interval 6. Venter light brownish yellow, sterna sometimes darker; legs yellow (Fig. 1).

Head microsculptured, deeply and irregularly punctate, punctures sometimes almost confluent in frontal depressions, on the frons separated by 3–5 their diameters, towards the base of head, by 1–2 diameters. Clypeus with a few punctures at lateral margins. Eyes large and bulging, with numerous strong setae at posterior margin. Second supraocular seta located just anterad the posterior margin of eye. Temples smooth, 2.4 times shorter than length of eye. Scape with a very long seta and a few long thin setae; pedicel with a band of apical setae; antennomere III with scattered setae on apical half. Antennae pubescent from the mid-length of antennomere IV.

Pronotum shinier than head, microsculptured, cordate, 1.08–1.11 times as wide as head, 1.2–1.25 times as wide as long, widest just behind the marginal setae. Anterior margin straight, anterior angles effaced, sides slightly sinuate towards posterior angles, which are rectangular or slightly acute. Disc sparsely irregularly punctate, somewhat less densely than head; punctures at lateral margins confluent, surface rugose. Disc convex to lateral margins, which are very narrow. Posterior pore right in front of angle. Basal grooves shallow, rugose, confluently punctate. Pronotal base extended in a rounded median lobe. Furrow short and shallow.

Each interval with a row of setiferous pores along the middle from base to apex. Setae as long as the width of interval 2. Microsculpture almost isodiametric. Apices slightly sinuate. Striae deep, punctate. Intervals weakly convex at base, flat at apex.

Legs brownish yellow. Tarsomere V with 4 pairs of ventral setae. Propleuron smooth, even, mes- and metepisterna strongly microsculptured. Claws with 4 teeth, 3 relatively long and one (basal) minute. All abdominal sterna with long pubescence.

Aedeagus – Fig. 28. Internal sac without apparent spicules or microtrichial patches.

##### Variation.

Varies in size, ventral colour, and the extent of dark elytral pattern (may be reduced).

##### Comments.

Types collected in “Samarkand" (now Uzbekistan) and near Shahrud (n. Iran) (Solsky, 1874: 35).

##### Distribution.

Afghanistan, Iran, Kyrgyzstan, Kazakhstan, Tajikistan, Turkmenistan, Uzbekistan.

#### Singilis cingulatus

(Gebler, 1843)

http://species-id.net/wiki/Singilis_cingulatus

Dromius cingulatus Gebler 1843: 37.
Agatus cingulatus : Gebler 1859: 319.
Glycia cingulata : Solsky 1874: 34.
Glycia cruciger Heyden 1889: 327.
Glycia fasciata Motschulsky 1844: 42 (Lectotype in ZMM)
Agatus afghanus  a. *nigripes*Jedlička 1961b: 163 syn. n.
Singilis jakeschi Jedlička 1967: 102 syn. n.

##### Material examined.

HOLOTYPE, 1 golden foil circle, 1 handwritten label: “cingulatus Gebl. Sibir. Or." (indeed = Ala-kul') (ZIN); AFGHANISTAN: *Singilis jakeschi* HOLOTYPE Jedlička, N Afganistan, prov. Heart prov., coll. O. Jakeš, Bala Murghab, 30.VI–2.VII.1964, 470m, (37) (1♂, MMBC); N Afganistan, prov. Heart prov., Bala Murghab, 4.XI.1964, 470m, coll. O. Jakeš (102) (1 ex., MMBC); same place, 31.X.1964, (97) (3 ex., MMBC); same place, 2.XI.1964, (99) (1 ex, MMBC); same place, 10–13.VII.1964, (100) (1 ex, MMBC); same place, 3.XI.1964, (101) (1 ex, MMBC); N Afganistan, prov. Heart prov., Akaza-i, 3.XI.1964, 450m (100) (1 ex, MMBC); N Afganistan, Prov. Mazar-i-Sharif, Mazar-i-Sharif, 15–30.XI.1964, 365m, coll. O. Jakeš (106) (2 ex, MMBC); *Agatus afghanus* a. *nigripes* det. Ing. Jedlička HOLOTYPE red label; J.Klapperich, Umgeb. v. Kabul, 1740 m., 20.III.53, O. Afghanistan (1♂, NMPC); COTYPE of *Agatus afghanus* a. *nigripes* Afganistan, Douchi (1♀, NMPC). KAZAKHSTAN: S Kazakhstan, Karatau Mt. Rng., 40 km N Igilik vill., Kurkal, N43°47'003", E68°03'138", 543m, 8.V.2010, light trap, Ivanov A.V. leg. (16♂♂ 19♀♀, AAC); Syr Darja, Turkest. (1 ex., NMPC); Turkestan, Auli-Ata, C.Aris (1♂ 1♀, MNHN). TURKMENISTAN: Kaahkinskij raj., 28.IV–25.V.1994, A. Kalninsh leg. (1♂, DUBC); Turcmenia, Leder, Reitter; second label: *Singilis jakeschi* Jedl. det. Ing. Jedlička (1♂, NMPC). UZBEKISTAN: COTYPE of *Agatus afghanus* a. *nigripes*, Buchara (1♂, NMPC).

##### Diagnosis.

This species is most similar to *Singilis flavipes*, with diagnostic differences listed under that species.

##### Redescription.

Length 4.5–5.2 mm. Body elongate, head and pronotum red-brown, elytra black with red-brown basal band and two rounded preapical spots, often confluent. Femora and tibial apices piceous to black (Figs 2–3).

Head microsculptured, deeply irregularly punctate, punctures sometimes almost confluent in frontal depressions, on the frons separated by 3–5 times their diameters, towards the base of head, by 1–2 diameters. Clypeus with a few punctures at lateral margins. Eyes large and bulging, with numerous moderately long setae at posterior margin. Second supraocular seta located just anterad the posterior margin of eye. Temples smooth, 4.2 times shorter than length of eye. Scape with a very long seta and a few long thin setae; pedicel with a band of apical setae; antennomere III with scattered setae on apical half. Antennae pubescent from the mid-length of antennomere IV.

Pronotum shinier than head, microsculptured, cordate, 1.07 times as wide as head, 1.25–1.31 times as wide as long, widest just behind the marginal setae. Anterior margin straight, anterior angles effaced, sides slightly sinuate towards posterior angles, which are rectangular or slightly acute. Disc sparsely irregularly punctate, somewhat less densely than head; punctures at lateral margins confluent, surface rugose. Disc convex to lateral margins, which are very narrowly explanate. Posterior pore right in front of angle. Basal grooves shallow, rugose, confluently punctate. Pronotal base extended in a rounded median lobe. Furrow shallow and short.

Each interval with a row of setiferous pores along the middle from base to apex. Setae as long as the width of interval 2. Microsculpture almost isodiametric. Apices slightly sinuate. Striae deep, punctate. Intervals weakly convex at base, flat at apex.

Femora and apical parts of tibiae piceous to black. Tarsi sparsely pubescent, with long dorsal setae. Tarsomere V with 4 pairs of ventral setae. Propleuron rugose, mes- and metepisterna strongly microreticulate. Claws with 5 teeth. Abdominal sterna black, venter otherwise red-brown. Abdominal sterna with long pubescence.

Aedeagus – Fig. 29. Median lobe apex elongate, slightly downturned at narrowly rounded apex. Internal sac with two groups of large spines.

##### Variation

**.** Varies in size and colour: venter all-black to all-red, elytral dark pattern sometimes reduced, head and pronotum sometimes blackish. Pronotal sides sometimes sinuate towards base, in which case hind angles acute.

##### Comments.

The study of the holotype of *Singilis jakeschi* Jedlička, 1967, has shown that *Singilis jakeschi* is synonymous with *Agatus cingulatus* (Gebler, 1843). *Singilis jakeschi* was originally described based on a single female with reduced dark elytral pattern, taken from a large series of *Agatus cingulatus* collected in the same place, and determined by Jedlička as *Singilis afgana* and *Agatus afghanus* a. *nigripes*.

##### Distribution.

Afghanistan, Iran, Iraq, Israel, Kyrgyzstan, Kazakhstan, s. Russia, Tajikistan, Turkmenistan, Uzbekistan.

#### Singilis anthracinus

(Solsky, 1874)

http://species-id.net/wiki/Singilis_anthracinus

Glycia anthracina Solsky 1874: 36

##### Comments.

Species described from a single specimen labeled “Sibiria" (from collection of Eversmann) as similar to *Singilis cingulatus*, but differing by uniformly black body color. Body length 5 mm, length of elytra 3.5 mm, width 2.3 mm. Probably this species is just a color variation or melanic form of *Singilis cingulatus*. The type was supposed to be in ZIN but we were unable to locate it. To make a final decision about the taxonomic status of this taxon it is necessary to study the type and additional material.

#### Singilis amoenulus

(Semenov, 1889)

http://species-id.net/wiki/Singilis_amoenulus

Glycia amoenula Semenov 1889: 400

##### Material examined.

Syntypes: Turkestan, W. Balassoglo leg. (1♂ 1♀, ZIN); TURKMENISTAN: SW Turkmenistan, Beki-Bent, 15.V.1952, Romadina leg. (1♂, ZIN); Kara-Bogaz, 40 km N Kizyl-Arvat, 14.IV.1952, Il'ichev leg. (1 ex., ZIN); W Kopetdag, 15 km S Iskender, 15.V.52, Kir'janova leg. (1♀, ZIN); SW Turkm., vall. of Divan, 15 km W Chat, 4.V.1952 (1♀, ZIN); Turkm., 13 km S Kizil-Arvat, 25.IV.52 (1♂, ZIN); Chardzhou, Amu-Dar'ja riv., 8.VIII.1910, N. Androsov leg. (2♂♂ 2♀♀, ZIN); Kushka, Zakaspiisk reg., 16.VI.1912, leg. V. Kozhanchikov (2♂♂, ZIN); Kushka, 29.VII.1910 (3♂♂ 5♀♀, ZIN); Turkm., Eroilan-Duz, 1.V.1968, G. Medvedev leg. (1♀, ZIN); Tamdytau, Aktau, 4.V.1965, Medvedev leg. (1♂, ZIN); Turkm., Kara-Kala, Sjumy, IX.1931 (1♂, ZIN); Turkmenia (1♀, NMPC). UZBEKISTAN: Termez, Buhara, 3.VII.1912, Kirichenko leg. (1♀, ZIN).

##### Diagnosis.

Instantly recognizable by the combination of cordate pronotum, elongate and flat elytra, intervals with single uninterrupted row of conspicuous setiferous pores bearing long setae. In sympatric *Singilis solskyi* intervals of elytra with 2–3 irregular and dense rows of big setiferous pores.

##### Redescription.

Length 5.9–6.2 mm. Red-brown, with wide piceous postmedian band on elytra (Fig. 4).

Head smooth, very distinctly microsculptured, finely punctulate, punctures separated by 2–4 diameters. Clypeus with few punctures at base. Eyes normal, with 3–5 setae at posterior margin. Second supraocular seta located just anterad the posterior margin of eye. Temples long, smooth. Scape with a very long subapical seta and several short setae; pedicel and antennomere III each with a single band of apical setae. Antennae pubescent from the mid-length of antennomere IV.

Pronotum cordate, smooth, 1.1 times as wide as head and 1.27 times as wide as long, widest right behind marginal setae. Anterior margin straight, anterior angles effaced, sides strongly sinuate towards base. Posterior angles acute, prominent. Lateral margin narrow. Discal punctation sparse and shallow, somewhat sparser than on head. Posterior pore right in front of angle. Lateral and basal setae long. Basal grooves punctiform. Pronotal base rugosely punctate, extended in a rounded median lobe. Microsculpture as on head.

Each interval of elytra with a single regular row of long sparse setae, interval 8 with two rows of setae. Setae as long as the width of interval 3 at its widest. Pores deep, punctiform, separated by three diameters. Microsculpture of elytra and scutellum irregularly polygonal, shallower than on head and pronotum. Elytral apices weakly obliquely sinuate, rounded at suture. Striae deep and punctate. Intervals almost flat throughout.

Legs pale brownish. Tarsomere V with 4 pairs of ventral setae, two basal pairs very short. Claws with 3 denticles on basal half. Venter entirely brownish yellow. Episterna smooth. All abdominal sterna with long pubescence.

##### Distribution

**.** Kazakhstan, Turkmenistan, Uzbekistan.

#### Singilis solskyi nom. n.

Glycia bicolor Solsky 1874: 35 [nec Rambur 1837]
Agatus (Phloeozetoeus) taschkensis Jedlička 1961b: 163

##### Material examined.

Holotype: ♀, red label: *Singilis taschkensis* sp. n., det. Ing. Jedlička, red label: Typus, White label: Tshingan, Taschkent (NMPC); AFGHANISTAN: Farah, W Sindand, 900m, 3.XI.1970, Kabakov leg. (2 ex., ZIN). IRAN: NO Iran, W v. Meshed, 21.VI.1963, Kasy & Vartian (1 ex., NHMW). KAZAKHSTAN: confluence of Arys riv., Syr-Daria, Chimkent, 21.V.1898 (1 ex., ZMM); Syr-Daria reg., pass near Baigakum, Dzhulek, D. Glazunov leg. (5 ex., ZMM); Baigakum (1 ex., ZMM); S Kazakhstan, Karatau Mt. Rng., 40 km N Igilik vill., Kurkal, N43°47'003", E68°03'138", 543m, light trap, 8.V.2010, Ivanov A.V. leg. (3♂♂ 3♀♀, AAC). TAJIKISTAN: Shaartuz env., Chiligor-Chashme, 19.IV.1960, Lopatin leg. (1 ex., ZMM); Aruk-tau Mt. Rng., Gandzhina, 1000–1200m, 24.IV.1962, Kryzhanovskii leg. (1 ex., ZMM). TURKMENISTAN: Kushka, Zakaspiisk reg., 10.VI.1912, Kozhanchikov leg. (2 ex., ZMM); Badhyz, Eroulanduz, 12.IV.80, H. Atamuradov leg. (1 ex., ZMM); SE Turkmenistan, Gaz-Giadyk mt.rng., Akar-Cheshme, 6.IV.1977, Dolin leg. (1 ex., ZMM); Tedzhen' riv., Akar-Cheshme, Zakaspiisk reg., 5.V.1895 (1 ex., ZMM); Imam-Baba (6 ex., ZMM); SE Turkmenistan, Giaz-Gadyk Mt. rng., Akar-Cheshok, 6.IV.1977, Dolin leg. (1♂, ZIN); Turkmenia, Zulphager Mts. Rg., 38 km SE Pulikhetun, 13.IV.93 (1♂, AAC). UZBEKISTAN: Samarkand env., Chahryn, 1.IV.1912 (1 ex., ZMM); Samarkand (2 ex., ZMM); Kugitantau, led mines, 14.IV.1959, Medvedev leg. (1 ex., ZMM); Buhara, Dzham, 22.V.06, G. Suvorov leg. (1 ex., ZMM); Taschkent (1 ex., ZMM); Guzar-Tengi, Horan, Buhara, 28.IV.1987, Kaznakov leg. (1 ex., ZMM); Zerbant, W Buhara, 27.IV.1912 (1 ex., ZMM); Buhara, Kizil-Al'ma, 17.V.1910, Zarudnyi leg. (1 ex., ZMM); Turcest. Taschkent (1 ex., ZMM); Turcest., Tashkent (2 ex., ZIN). LOCALITY NOT IDENTIFIED: Turkestan, Sahsar, 1892 (1 ex., ZMM).

##### Diagnosis.

This species is most similar to the sympatric *Singilis makarovi*, new species. This species shares with *Singilis makarovi* the presence of 2–3 irregular and dense rows of big setiferous pores on all intervals of elytra, bearing long setae. The two species can be diagnosed easily by the propleura, wavy rugate in *Singilis solskyi*, smooth in *Singilis makarovi*.

##### Redescription.

Length 6.8–8.0 mm. Head, pronotum, basal third of elytra, thorax, legs, and antennae red-brown; suture, apical 2/3 of elytra, and abdominal sterna black (Fig. 5).

Head microsculptured, deeply irregularly punctate, punctures near eyes sometimes almost confluent, on the frons separated by more than three diameters and on the vertex by 1–2 diameters. Clypeus sparsely punctate. Eyes large and bulging, with numerous strong setae at posterior margin. Second supraocular seta located just anterad the posterior margin of eye. Temples smooth. Scape with a very long seta and a few short thin setae scattered throughout; pedicel with a band of apical setae; antennomere III with numerous setae on apical half. Antennae pubescent from the basal third of antennomere IV.

Pronotum shinier than head and elytra, 1.18 times as wide as head, 1.34 times as wide as long, widest in front of marginal setae. Anterior margin straight, anterior angles effaced, sides very broadly and evenly rounded, slightly sinuate towards posterior angles, which are rectangular. Disc shiny, very sparsely and irregularly punctate, punctures separated by 2–5 diameters, transversely confluent towards lateral margins. Lateral margins narrow, flat towards base. Posterior pore right in front of angle. Basal grooves shallow, rugose, confluently punctate. Pronotal base extended in a rounded median lobe. Furrow fine and reduced. Microreticulation faint.

Elytra pubescent. Each interval with 1–2 irregular rows of setiferous pores from base to apex. Setae as long as mesotarsomere IV. Interval 7 flat, as wide as adjacent intervals. Microsculpture polygonal, deep and irregular, same as on head. Apices slightly sinuate. Striae slightly punctate, shallower on disc and at apices. Intervals slightly convex at base, flat at apex.

Tarsomere V with 5 pairs of ventral setae. Propleura rugose. Mes- and metepisterna smooth. Claws with 5 teeth, basal teeth very small. All abdominal sterna pubescent; pubescence long and dense, as on elytra.

Aedeagus – Fig. 30. Median lobe apex elongate, slightly downturned at narrowly rounded apex. Internal sac with one large and compact spicular field made up of mid size spines.

##### Distribution.

Afghanistan, Kazakhstan, Kyrgyzstan, Tajikistan, Turkmenistan, Uzbekistan.

##### Comments.

This species was described as *Glycia bicolor* (Solsky, 1874) (Types: Samarkand, Karasu, near Katty-Kurgan, 25.IV.1869; and Ulus riv., 1869). Now that *Agatus* has been synonymized with *Singilis*, *Singilis bicolor* (Solsky, 1874) has become a junior homonym of *Singilis bicolor* Rambur, 1837, and should be replaced. We propose the name *solskyi* nom. n.for *Singilis bicolor* (Solsky, 1874), nec Rambur 1837.

##### Name derivation.

Named after the Russian entomologist Semyon Solsky who first described this species.

#### Singilis makarovi sp. n.

urn:lsid:zoobank.org:act:CE03FEA8-980B-4D45-9643-334A88A6513C

http://species-id.net/wiki/Singilis_makarovi

##### Material examined.

Holotype: ♂, Tajikistan, Petr 1 mt. rng., western Sangvor, Luli-Harvi, deciduous forest, 23.VIII.1975, leg. V. Yanushev (MPU). Paratype: ♀, same label (MPU).

##### Diagnosis.

This species is most similar to *Singilis solskyi*, with diagnostic differences listed under that species.

##### Description.

**Figures 6–7.**
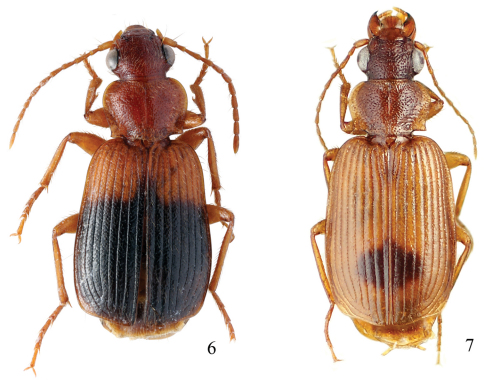
Habitus of *Singilis*: **6** *Singilis makarovi* sp. n., Paratype **7** *Singilis timidus* sp. n., Paratype (Iran, Lorestan).

Length 6.0 mm. Head, pronotum, and legs red-brown. Venter red-brown, except black abdominal sterna. Elytra black, with basal third red-brown (Fig. 6).

Head densely punctate, very distinctly microsculptured and pubescent; pubescence as long as on pronotum and elytra, and equal to the width of the sutural elytral interval. Punctures near eyes sometimes almost confluent, on the frons separated by over twice their diameter. Clypeus smooth, very distinctly microsculptured. Eyes large and bulging, with 4–5 long setae at posterior margin. Second supraocular seta located just anterad the posterior margin of eye. Temples very short, smooth. Scape with a very long subapical seta and several more, rather long, setae; pedicel with a band of setae; antennomere III with two bands of apical setae. Antennae pubescent from the basal third of antennomere IV.

Pronotum evenly setose, 1.22 times as wide as head, 1.36 times as wide as long, widest near marginal setae. Anterior margin weakly emarginate, anterior angles effaced, sides broadly evenly rounded, sinuate towards posterior angles, which are acute and protrude as minute denticle. Punctation uniform and deep, somewhat shallower than on the head, especially on the disc of pronotum. Lateral margin moderately explanate in basal half only. Posterior pore right in front of angle. Basal grooves shallow, small, punctiform. Pronotal base extended in a rounded median lobe. Furrow fine, not reaching apical and basal margins. Microsculpture as on head.

Elytra: All intervals with confused punctation, conspicuous polygonal microsculpture and long, pale pubescence. Apices weakly obliquely sinuate, rounded at suture. Striae deep and crenulate. Intervals almost flat on disc, slightly convex at base and flat towards apex. Scutellum smooth.

Tarsomere V with 4–5 pairs of ventral setae. Claws with 5 teeth, of which apical three very long. Prosternum, propleura and mesepisterna smooth. Abdominal sterna pubescent.

Aedeagus – Fig. 31. Aedeagal median lobe moderately broad, ventral margin straight nearly to apex. Apex broad, slightly downturned and expanded near broadly rounded tip. Internal sac with big size spines.

##### Name derivation.

Named after Dr. Kirill V. Makarov.

##### Distribution.

Tajikistan.

#### Singilis kabakovi sp. n.

urn:lsid:zoobank.org:act:BA3EF765-DA34-4DEB-B5B7-DF59061031A6

http://species-id.net/wiki/Singilis_kabakovi

##### Material examined.

Holotype: ♂, Afghanistan, Herat, NW Adraskan, 20.XI.1971, leg. Kabakov (ZIN). Paratypes: Afghanistan, Ghazni, W Moqur, 2300 m, 11.IX.1972, leg. Kabakov (1♀, ZIN); Iran, Khorasan, Torbat-e-Heydariyeh, 5 km S Zharf, 14.V.2007, leg. A. Anichtchenko (1♂, AAC); small green rectangle and a white label “Christoph collection" (in Russian) meaning Schahrud, Persia 1870–1873 (1♀, ZIN).

##### Diagnosis.

**Figures 8–11.**
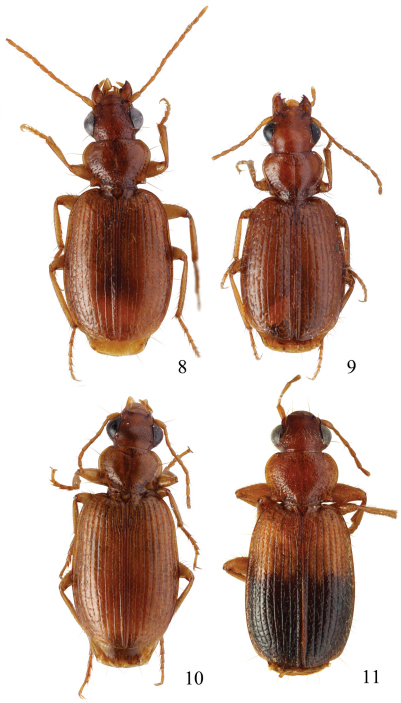
Habitus of *Singilis*: **8** *Singilis kabakovi* sp. n., Paratype (Iran, Khorasan, E Torbat e Heidarieh, 5 km S Zharf) **9** *Singilis kolesnichenkoi* sp. n., Holotype **10** *Singilis klimenkoi* sp. n., Paratype **11** *Singilis timuri* sp. n., Holotype.

This plus *Singilis kolesnichenkoi* sp. n., *Singilis klimenkoi* sp. n. and *Singilis timuri* sp. n. constitute a quartet of species consisting of small-bodied beetles, with convex, subovate elytra and long pubescence (Figs 8–11). *Singilis kabakovi* alone has the elytra with weak, diffuse, grey postmedian band, sometimes reduced to sutural spot and intervals 2, 4 and 6 without setiferous pores.

##### Description.

Length 5.2–5.8 mm. Red-brown, with postmedian sutural infuscation on elytra (Fig. 8).

Head smooth, microsculptured, uniformly punctate, punctures separated by 1–2 their diameters. Clypeus impunctate. Eyes moderately convex, with a few relatively long setae at posterior margin. Second supraocular seta located just anterad the posterior margin of eye. Temples short and smooth. Scape with a very long seta at 2/3 of its length and a few thin setae towards the apex; pedicel with a single, apical band of setae; antennomere III with a few additional setae towards apex. Antennae pubescent from the basal fourth of antennomere IV.

Pronotum smooth, shinier than head, 1.14 times as wide as head, 1.2 times as wide as long, widest at marginal setae. Anterior margin straight, anterior angles slightly prominent, sides very broadly and evenly rounded, conspicuously sinuate towards posterior angles, which are acute. Punctation irregular, sparser and shallower than on the head. Explanate lateral margin rapidly widened from apex, broad and flat to slightly reflexed at base. Lateral and apical setae very long. Posterior pore right in front of angle. Basal grooves punctate. Pronotal base extended in a rounded median lobe. Furrow variable. Microsculpture subtle, slightly transversely polygonal.

Elytra infuscate behind middle, infuscation may reach just one or two innermost intervals, or extend to lateral margins. Intervals 1, 3, 5, 7, 8 pubescent throughout, and interval 6 pubescent in apical half, each with an irregular row of large setiferous pores. Setae as long as the width of interval 4 at its widest. All intervals convex at basal third and slightly convex from there to apex. Interval 7 convex from base to mid-length. Microsculpture deep, irregularly polygonal. Apex slightly sinuate. Striae deep, punctate, shallower at apex.

Legs brownish yellow. Tarsomere V with 3 pairs of ventral setae. Mes- and metepisterna smooth. Claws with 4 moderately long teeth. Venter evenly brownish yellow. All abdominal sterna pubescent, pubescence as long as on elytra.

Aedeagus – Fig. 32.

##### Name derivation.

Named after Oleg Kabakov, a famous Russian coleopterist.

##### Distribution.

Afghanistan, Iran.

#### Singilis kolesnichenkoi sp. n.

urn:lsid:zoobank.org:act:8E48510D-B30F-4F18-BAAD-8C3EA324DE80

http://species-id.net/wiki/Singilis_kolesnichenkoi

##### Material examined.

Holotype: ♀, Iran, Kerman, Sirjan, 8 km N Balvard, 11–12.V.2007, leg. A. Anichtchenko (ZIN).

##### Diagnosis.

Among the species consisting of small-bodied beetles, with convex, subovate elytra and long pubescence, *Singilis kolesnichenkoi* is diagnosable by the smooth head, and almost smooth, cordate pronotum; rows of setiferous punctures on intervals 3–5 widely interrupted; setiferous pores on all intervals large; body coloration uniformly red brownish.

##### Description.

Length 4.6 mm. Uniformly red-brown, with venter and legs paler (Fig. 9).

Head smooth, distinctly microsculptured, very sparsely minutely punctulate, punctures sometimes barely visible. Clypeus and labrum distinctly microsculptured. Eyes moderately large, each with 3 long setae behind. Second supraocular seta located immediately basad the posterior margin of eye. Temples long, smooth. Scape with a very long subapical seta and several small setae; pedicel and antennomere III each with a single band of apical setae. Antennae pubescent from the mid-length of antennomere IV.

Pronotum smooth, cordate, 1.07 times as wide as head, 1.3 times as wide as long, widest just behind marginal setae. Anterior margin straight, anterior angles effaced, sides broadly and regularly rounded, slightly sinuate towards base, posterior angles obtuse. Disc very sparsely punctate. Anterior margin with a few big punctures, basal part with large punctation reaching along lateral margins 1/3 of pronotal length. Lateral margin narrowly explanate from apex, slightly wider at base. Posterior pore right in front of angle. Basal grooves shallow. Pronotal base extended in a rounded median lobe. Furrow long and fine. Microsculpture as on the head.

Elytra suboval, convex, with strong polygonal microsculpture. Each interval with a series of very large setiferous pores in a single row. In the discal part of intervals 2–5 pores may be smaller or rows interrupted. Setae as long as the combined width of intervals 1 and 2. Scutellum smooth and shiny, without microsculpture. Elytral apices truncate, weakly obliquely sinuate, rounded at suture. Striae crenulate at base, finely punctate elsewhere. Intervals slightly convex.

Legs pale brownish yellow. Tarsomere V with 2 pairs of short ventral setae. Claws with 4 minute denticles near base. Abdominal sterna smooth, shiny, with sparse short setae.

**Name derivation:** Named after my friend, Dr. Kirill Kolesnichenko.

##### Distribution.

Iran.

#### Singilis klimenkoi sp. n.

urn:lsid:zoobank.org:act:D6BA6713-9986-4CEF-8A12-31A49154979C

http://species-id.net/wiki/Singilis_klimenkoi

##### Material examined.

Holotype: ♂, SE Iran, Sistan, 100 km SE Zahedan, Tamin, 2100m, 3.V.2006, leg. A. Klimenko (ZIN); Paratype: ♂, same label (AAC).

##### Diagnosis.

Among the quartet of species consisting of small-bodied beetles, with subovate elytra and long pubescence (Figs 8–11), easily distinguishable by the single uninterrupted row of widely spaced setiferous pores on all elytral intervals and uniformly yellow brownish coloration.

##### Description.

Length 4.7–5.1 mm. Uniformly yellow-brown, legs paler (Fig. 10).

Head smooth, very distinctly microsculptured, finely punctulate, punctures separated by 2 to 4 diameters. Clypeus impunctate. Eyes large and bulging, with 2–3 short setae at posterior margin. Second supraocular seta located just anterad the posterior margin of eye. Temples short, smooth. Scape with a very long subapical seta and several short setae; pedicel and antennomere III each with a single band of apical setae. Antennae pubescent from mid-length of antennomere IV.

Pronotum smooth, 1.08 times as wide as head, 1.38 times as wide as long, widest right behind marginal setae. Anterior margin straight, anterior angles effaced, sides almost straight basad the widest point. Posterior angles obtuse. Lateral margin narrow, 2–3 transverse wrinkles at base. Discal punctation sparse and shallow, somewhat sparser than on head. Posterior pore right in front of angle. Lateral and basal setae long. Basal grooves punctiform. Pronotal base extended in a rounded median lobe. Furrow deep and short. Microsculpture as on head.

Elytra shinier than head and pronotum. Each interval with a single irregular row of sparse setae; setae longer than the width of interval 3 at its widest. Setiferous pores large and flat, sparser on intervals 3 and 5 than elsewhere. Microsculpture polygonal, more delicate than on head and pronotum. Apices weakly obliquely sinuate, rounded at suture. Striae very finely punctate. Intervals slightly convex at base and flat at apex. Scutellum microsculpture as on elytra.

Tarsomere V with 4 pairs of ventral setae, two basal pairs very short. Claws with 3 denticles in basal half. Venter brownish yellow throughout. Episterna smooth. Abdominal sterna with long pubescence.

Aedeagus – Fig. 33. Aedeagal median lobe moderately broad, ventral surface straight at midlength, apical fourth of lobe slightly downturned, tip long. Internal sac with compact field of large spines.

##### Name derivation.

Named after Alexei Klimenko who collected the specimens.

##### Distribution.

Iran.

#### Singilis timuri sp. n.

urn:lsid:zoobank.org:act:323A6D0C-1849-4218-BB17-48ECCE673ADB

http://species-id.net/wiki/Singilis_timuri

##### Material examined.

Holotype: ♂, Aman-Kutan, merid. versus, ab Samarkand, 14.V.65 (ZMM).

##### Diagnosis.

Among the quartet of small-bodied species, with convex, subovate elytra and long pubescence (Figs 8–11), *Singilis timuri* is diagnosable by the black apical half of elytra.

##### Description.

Length 5.1 mm. Red-brown with apical half of elytra black; anterior margin of the dark area blurry and perpendicular to suture (Fig. 11).

Head sparsely feebly punctate on the frons and between eyes, with punctures separated by 4 to 7 diameters, deeply and densely punctate elsewhere, with punctures separated by their diameter towards head base. Head and clypeus very distinctly microsculptured throughout. Clypeus smooth. Head pubescent, pubescence as long as on pronotum and elytra and as long as width of second interval. Eyes moderately large and bulging, with 4–5 long setae at posterior margin. Second supraocular seta located just anterad the posterior margin of eye. Temples short, smooth. Scape with a very long subapical seta and several more, rather long, setae; pedicel with a band of setae; antennomere III with two apical bands of setae. Antennae pubescent from the basal third of antennomere IV.

Pronotum 1.22 times as wide as head, 1.33 times as wide as long, widest near marginal setae. Anterior margin straight, anterior angles effaced, sides broadly and regularly rounded, sinuate at base, posterior angles rectangular. Punctation confused and coarse, punctures large, shallower and larger towards the furrow and anterior angles. Disc convex, uniformly setose. Lateral margin slightly explanate in basal half only. Posterior pore right in front of angle. Basal grooves flat, densely punctate. Pronotal base extended in a rounded median lobe. Furrow deep, almost reaching anterior margin and ended short of the base by length of antennomere III. Microsculpture as on head.

Elytra subovate, with polygonal microsculpture. Each interval with a series of large setiferous pores in a single irregular row. Scutellum smooth. Elytral apices truncate, weakly obliquely sinuate, rounded at suture. Striae deep and slightly crenulate. Intervals slightly convex.

Legs red-brown. Protarsomere IV small and narrow. Tarsomere V with 4 pairs of ventral setae. Claws with 3 long apical teeth and one tiny denticle. Abdominal sterna black, pubescent throughout. Prosternum, propleura and mesepisterna smooth.

Aedeagus – Fig. 34. Aedeagal median shaft slightly arcuate between basal bulb and elongate, narrow apex with tightly rounded tip. Internal sac with few large spines.

##### Name derivation.

Named after Timur (Tamerlane), the fourteenth-century conqueror of Asia.

##### Distribution.

Uzbekistan.

#### Singilis discoidalis

(Mateu, 1986)

http://species-id.net/wiki/Singilis_discoidalis

Phloeozeteus discoidalis Mateu 1986: 200

##### Material examined.

EGYPT: Aegyptus, Faggala (1♀, NMPC); Le Caire (1♀, MNHN). ISRAEL: Arava-Tal, S Zofar, 18.II.1987, 0 m, leg. Schawaller & Schmalfuss (1♂, SMNS). YEMEN: Lahj, X.2000, malaise trap, leg. A. v. Harten & A. Sallam (1♂ 1♀, SMNS).

##### Diagnosis.

Most similar in overall appearance and small size to *Singilis turcicus*, but with pronotum strongly transverse and pronotal punctures spaced twice as far apart as those on the head.

##### Redescription.

Length 4.5–4.8 mm. Winged. Ferrugineous or testaceous red with a dark preapical sutural spot reaching interval 4 or 5 (Fig. 16).

Head obtuse, rather wide, slightly convex to flat between the eyes, with numerous large and deep punctures (mixed with wrinkles near eyes) that become sparser towards the neck. Frons without punctures. Eyes large and convex.

Pronotum punctate, slightly wrinkled, transverse, subconvex, considerably broader than head, strongly rounded in front, with anterior angles effaced. Sides rounded, with a long sinuation in front of posterior angles, which are acute. Pronotal margin broadly explanate, basal impressions fairly deep. Pronotal base projected at the middle, median furrow moderately wide and deep.

Elytra approximately 1/3 longer than broad, sides subparallel, almost obliquely truncate at apex which is weakly sinuate. Striae, including the scutellar stria, fairly deep and finely punctate; intervals weakly convex, with two pores on interval 3.

Microsculpture strong, especially on forebody where the cells are small and rather isodiametric, but cells become more transverse towards the sides of pronotum. Elytra with finer microsculpture of larger, subquadrate cells, and thus appearing shinier than the forebody.

In male, protarsomeres I–III slightly dilated.

Aedeagus – Fig. 35. Aedeagal median lobe elongate, straight, with notch in middle, emphasized by a thickening in the manner of a sagittal crest. Tip of aedeagus slightly curved to right. Internal sac with small spicular field made up of small spines.

##### Distribution.

Egypt (country record), Israel (country record), Saudi Arabia, Yemen.

#### Singilis turcicus

(Jedlička, 1963a)

http://species-id.net/wiki/Singilis_turcicus

Phloeozetteus turcicus  Jedlička, 1963a: 6

##### Material examined.

Holotype, ♀, red label: *Phloeozetteus turcicus* sp. n. det. Ing. Jedlička; white label: Türkei, Marasch, 12.V.60, leg. Seidenstücker (ZSM). ARMENIA: Syunik prov., E Meghri, Artsvakar gorge, 650m N38°55', E46°16', light, 8.VI.2007, Kalashian leg. (1♂, IZEC). IRAN: Chaharmahal-va-Bakhuyari prov., 10 km E Chaman Goli, 7–8.VI.2008, 3500m, Anichtchenko A. leg. (1♂, AAC); Fars, near Sarvestan, 8.V.2007, Anichtchenko A. leg. (1♂, AAC); Fars, 10 km SW Kharameh, 31.V.2008, Anichtchenko A. leg. (1♂, AAC); Kerman, Qohrud mts., 10 km E Korin, 3500m, 13.V.2007, Anichtchenko A. leg. (2♂♂, AAC); Kohgiluyeh-va-Boyer Ahmad, 20 km SW Yasuj, 5–6.V.2007, Anichtchenko A. leg. (1♀, AAC); SW Iran, Fars, Sivand NE Shiraz, 1770 m, 3008N 5255E, lux, 15.VII.2004, M. Rejzek leg. (3♂♂ 1♀, DWWC); W Iran, Lorestan, 25 km NWW Dorud, 1874m, 3333N 4853E, lux, 8.VII.2004, M. Rejzek leg. (1♂, DWWC); Kerman env., Deh Bala, 14.V.2003, Orszulik leg.(1♂ 1♀, KOC). ISRAEL: Jerusalem, 11.IX.1904 (1♀, NMPC).

##### Diagnosis.

This species is most similar to *Singilis discoidalis*, with diagnostic differences listed under that species.

##### Redescription.

**Figures 12–15.**
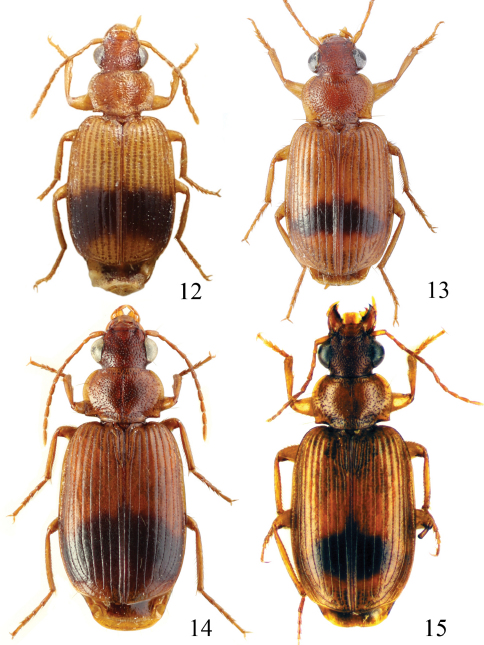
Habitus of *Singilis*: **12** *Singilis turcicus* (Jedlička, 1963a), Holotype **13** *Singilis turcicus* (Armenia, Syunik prov., E Meghri, Artsvakar gorge) **14** *Singilis mesopotamicus* Pic, 1901 (S Iran, 65km N Bandar Abbas) **15** *Singilis mesopotamicus* (Iran, Kerman, 15 km E Korin).

Length 4.3–5 mm. Yellowish red-brown, elytra with black pattern behind middle that varies from continuous band reaching lateral margins to small grey sutural spot (Figs 12–13).

Head microsculptured, deeply and uniformly punctate, punctures separated by about their diameter but denser in frontal depressions. Clypeus with few scattered punctures. Eyes large and bulging, with 4–5 very short setae at posterior margin. Temples short and smooth. Scape with a very long seta at 2/3 of its length and a few short thin setae towards the apex; pedicel with a band of apical setae; antennomere III with two bands of setae (at mid-length and at apex). Antennae pubescent from mid-length of antennomere IV.

Pronotum 1.21–1.27 times as wide as head, 1.42–1.45 times as wide as long, widest just behind the marginal setae. Anterior margin straight, anterior angles effaced, sides very broadly and regularly rounded, slightly sinuate towards posterior angles, which are rectangular or slightly acute, protruded as minute denticle. Discal punctation similar to that of head, but shinier due to more delicate microsculpture. Pronotum at apex and base rugulose and densely punctate. lateral margins narrowly explanate from anterior angle to lateral setae, then widened basally, broad and flat at base. Posterior pore anterad angle. Pronotal base extended in a rounded median lobe. Furrow short and fine.

Elytra in the middle 1.36–1.4 times as long as wide. Intervals 1, 3, 5 and 7 each with minute setae visible only in lateral view at high magnification, and two irregular rows of minute sparse setiferous pores from base to apex. Interval 7 convex in basal half and narrower than interval 6. Microsculpture polygonal. Apices slightly sinuate. Striae slightly punctate, shallower on disc and towards apex. Intervals slightly convex at base, flat at apex.

Legs brownish yellow. Tarsomere V with 5 pairs of ventral setae. Propleuron weakly rugose towards coxae. Mes- and metepisterna smooth. Claws with 4 teeth. Venter brownish yellow. All abdominal sterna with short pubescence, no longer than mesotarsomere IV.

Aedeagus – Fig. 36. Aedeagal median lobe slender, ventral surface straight at midlength. Endophallic spines quite uniform in size, less numerous than in *Singilis mesopotamicus*.

##### Variation.

Varies in body size and extent of dark elytral pattern (complete band to faint spot).

##### Distribution.

Armenia (country record), Iran (country record), Israel (country record), Turkey.

#### Singilis mesopotamicus

Pic, 1901

http://species-id.net/wiki/Singilis_mesopotamicus

Singilis (Phloeozeteus) plagiata  var. *mesopotamica*Pic 1901: 89
Singilis (Phloezetaeus) apicalis Jedlička 1956: 204, **syn. n.** (synonymy presumed; type of *Singilis mesopotamicus* Pic, 1901, unavailable).
Phloeozetus plagiatius mesopotamicus : Kabak 2003: 438

##### Material examined.

AFGHANISTAN: S Jalalabad, S Agam 2000 m, 23.XII.1970, Kabakov leg. (1♀, ZIN); 20 km NO Kabul, 2000m, 10.IV.1970 Kabakov (1♂ 3♀♀, ZIN); same locality, 15.IV.1971 (1♂, ZIN); Ghazni W Moqur 2300m, 5.X.1972, Kabakov (1♂ 2♀♀, ZIN); same locality, 11.IX.1972 (1♀, ZIN); Kondahar, Ghbargay 2000 m, 20.III.1973, Kabakov (2♀♀, ZIN); Ghor Saghar 2500 m, 14.VIII.1970 (1♂, ZIN); Heart, NW Adraskan 26.X.1971 (1♂, ZIN); same locality, 20.XI.1071 (1♀, ZIN). IRAN: Chaharmahal-va-Bakhuyari prov., 10 km E Chaman Goli, 7–8.VI.2008, 3500m, Anichtchenko A. leg. (2♂♂, AAC); Kerman, Qohrud mts., 10 km E Korin, 3500m, 13.V.2007, Anichtchenko A. leg. (2♀♀, AAC); S Iran, Kerman prov., Balt area, Korin v., 17–19.V.2008, A. Klimenko leg. (1♂, AAC); Kerman prov., 15 km E Korin, 2800–3300m, Kuh-e-Lalehzar mt., 17–19.V.2008, Anichtchenko A. leg. (1♂, AAC); Kerman, Sargad, 24–26.VI.1898, N. Zarudnyi leg. (1♀, ZIN); same locality, 25–27.VIII.1898 (1♂, ZIN); same locality, 30.IV.1901 (1♀, ZIN); Perse, Bender-Bouchir, Dr. Bussieres, 1905 (1♀, MNHN); S Iran, 65 km nördl. Bandar-Abbas, 30.III.1972, Exped. Mus. Vind. (1 ex, NHMW). IRAQ: Bagdad, coll. Kálalová (6♂♂ 4♀♀, NMPC); Bagdad, Irak, coll. Kálalová (4 ex., NMPC); Bagdad, Ex Musaeo H.W. Bates 1892, Museum Paris, 1952, Coll. R. Oberthür (2♂♂ 2♀♀, MNHN). PAKISTAN: NW Pakistan, prov. Swat, 71°90' L/ 35°70' B, Madyan, 1400 m, am Licht, 19.VI–4.VII.1971, leg. C. Holzschuh (2♂♂, NMPC); Northwest frontier Prov. Barseen, 35°21'42N, 73°12'42E, 900m, at light, No 26, 21.VII.1998, G. Csorba & L. Ronkay (1♂ 1♀, NHMW). TURKEY: Abanc-Sae, Preczmann, Aspock, Radda, 26.V.67 (1♀, NHMW).

##### Diagnosis.

Very similar in both external and aedeagal anatomy to *Singilis turcicus*. The widespread *Singilis mesopotamicus* somewhat varies in size, pronotal punctation, and elytral pattern. Endophallic anatomy in each species is rather uniform throughout its range, and the differences are minor and require further study. *Singilis turcicus* tends to be smaller, with elytra shorter and wider than in *Singilis mesopotamicus*, while the latter has a more transverse pronotum. In *Singilis turcicus*, the elytral spot is located immediately behind the middle, often restricted to innermost intervals, and never reaches elytral apices that are widely red-brown; while in *Singilis mesopotamicus* the spot is a bit farther posteriorly and usually reaches the apices (sometimes narrowly red-brown). Endophallic spine size is clearly different; small spines more numerous than in *Singilis turcicus*.

##### Redescription.

Length 5.0–6.3 mm. Yellowish brown, elytra behind the middle with piceous spot that may reach apical and lateral margins but sometimes not extended beyond interval 6 and often leaving elytral apices brownish red (Figs 14–15).

Head microsculptured and deeply irregularly punctate, punctures sometimes almost confluent in frontal depressions, on the frons separated by more than 3 diameters. Clypeus with a few punctures near base. Eyes large and bulging, with no setae at posterior margin. Second supraocular seta located just anterad the posterior margin of eye. Temples short and smooth. Scape with a very long seta and a few short thin setae; pedicel with a band of apical setae; antennomere III with two bands of setae (at mid-length and at apex). Antennae pubescent from the basal third of antennomere IV.

Pronotum pale yellow, sometimes shinier than head, 1.16–1.18 times as wide as head, 1.4–1.47 times as wide as long, widest just behind the marginal setae. Anterior margin straight, anterior angles effaced to slightly marked, sides very broadly and evenly rounded, slightly sinuate towards posterior angles, which are rectangular (normally) to subacute. Punctation, especially on disc, normally sparser and more delicate than on head; confluent and rugose towards lateral and posterior margins. Lateral explanate margin rapidly widens basad lateral setae, broad and flat at base. Posterior pore right in front of angle. Basal grooves shallow, punctate. Pronotal base extended in a rounded median lobe. Furrow variable. Microsculpture isodiametric.

Elytra 1.45–1.52 times as long as width in the middle. Intervals 1, 3, 5 and 7 each with minute setae and two irregular rows of pores from base to apex, often poorly visible. Even intervals may also bear a few setae in apical areas. Interval 7 convex and narrower than the adjacent intervals. Microsculpture nearly isodiametric. Apices slightly sinuate. Striae narrow and slightly punctate. Intervals slightly convex at base and flat behind the middle.

Legs brownish yellow. Metatarsomere V with 3–4 pairs of ventral setae. Propleura slightly wavy rugose towards sternum and coxae. Claws with 5 teeth. Venter brownish yellow throughout. All abdominal sterna pubescent; pubescence as short as on metatarsomere IV.

Aedeagus – Fig. 37. Aedeagal median lobe slender, ventral surface straight at midlength, apical fifth of lobe slightly downturned, tip long.

##### Variation.

Varies in size, elytral pattern (dark spot may be reduced), shape of posterior pronotal angles (usually acute and prominent but may be rectangular and less prominent). In almost all specimens from Afghanistan the apical spot reaches elytral apices, and the series of punctures in intervals 5 and 7 are shorter. Pakistani specimens have more convex elytral intervals.

##### Comments.

Jedlička (1956: 204) described *Singilis apicalis* from four syntypes (all NMPC): Afghanistan, Nuristan, Bashgultal (3♀♀); Asmar (1♀). Recent catalogues place this species in *Agatus* (Kabak 2003; Lorenz 2005).

*Singilis plagiatus mesopotamicus* Pic, 1901, was described from Baghdad. The original description says that the type is in coll. Pic (MNHN), but my attempts to locate it have been unsuccessful. However, 4 specimens of *Singilis apicalis* labeled “Bagdad" I've found in MNHN match the original description of *Singilis plagiatus mesopotamicus*. All other *Singilis* specimens (14 total) from Baghdad area I have examined were *Singilis apicalis*. Therefore I presume *Singilis apicalis* to be synonymous with *Singilis mesopotamicus*.

##### Distribution.

Afghanistan, Iran, Iraq (country record), Pakistan (country record), Turkey (country record), Turkmenistan.

#### Singilis fuscoflavus

(Felix & Muilwijk, 2009)

http://species-id.net/wiki/Singilis_fuscoflavus

Phloeozeteus fuscoflavus  Felix & Muilwijk, 2009: 125 (in Felix 2009)

##### Material examined.

HOLOTYPE: ♀, 5063 UAE, Hatta, 24°49'N, 56°07'E, 24–30.V.2006, light trap, leg. A. van Harten (UAEIC); Paratypes: 4578, same locality, 8–24.IV.2006 (2♀♀, RFC). OMAN: Oman bor., Prov. Batinah, Al-Jabal al-Ahdar mts., SE Rustaq, W Awabi, 430 m, Wadi Bani Awi, 23°20'0.13"N, 57°29'23.5"E, L. fang + L. fallen, 29–30.XII.2009 leg. Lehmann, Bittner & Stadie (1♂, APC).

##### Diagnosis.

This species can be recognised easily by the wide and small size body (4.8–5.4 mm), strongly transverse, very densely punctate pronotum and uniformly yellow pale body colour.

##### Redescription.

Length 5.1–5.6 mm. Pale brownish yellow, with no dark elytral pattern (Fig. 17).

Head pale yellowish red-brown, slightly darker than pronotum, very distinctly microsculptured and very coarsely and deeply irregularly punctate. Punctures sometimes almost confluent, sometimes separated by over two diameters. Clypeus twice as long as labrum. Eyes very large and bulging, with a few small setae at posterior margin. Second supraocular seta located just anterad the posterior margin of eye. Temples very short, smooth. Scape with a very long subapical seta and several rather long setae; pedicel and antennomere III each with a single band of apical setae. Antennae pubescent from the mid-length of antennomere IV. Antennomeres IV and V each slightly shorter than antennomere III. Antennomere V 2.5 times as long as wide at apex.

Pronotum 1.25 times as wide as head, 1.57 times as wide as long, widest just behind marginal setae. Anterior margin straight, anterior angles effaced, sides broadly and evenly rounded, slightly sinuate towards posterior angles, which are acute and form a minute denticle. Punctation coarse, irregular and deep, somewhat sparser than on head, especially on the disc. Punctures sometimes confluent, especially towards the sides of disc. Lateral explanate margin narrow at apex, rapidly widened basally. Posterior pore right in front of angle. Basal grooves shallow. Pronotal base extended in a rounded median lobe. Furrow very short and fine and does not reach anterior margin (by about the length of antennomere I) or the base. Microsculpture as on head.

Elytra generally concolorous with pronotum, with the apical third perhaps slightly darker. Intervals 1, 3, 5 and 7 with several minute, barely visible setae. Microsculpture lighter than on head and pronotum, so elytra seem shinier than head and pronotum. Scutellum with similar microsculpture. Elytral apices weakly obliquely sinuate, rounded at suture. Striae shallow, crenulate. Interval 8 convex over most of its length, slightly flattened towards apex; interval 7 convex only at shoulder; other intervals slightly convex near base and flat at apex.

Tarsomere V with 3 pairs of ventral setae. Claws with 4 teeth. Venter testaceous, abdomen slightly infuscated. Metathoracic process margined, with 4 long setae. Abdominal sterna pubescent; the last two also with scattered longer setae.

**Figures 16–19.**
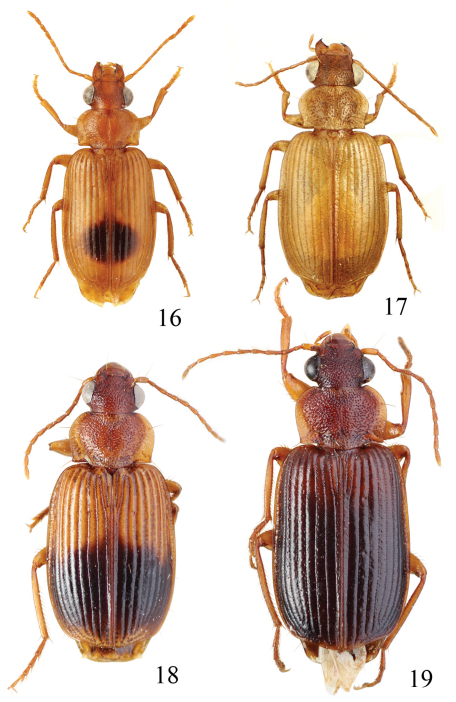
Habitus of *Singilis*: **16** *Singilis discoidalis* (Mateu, 1986) (Israel, Arava-Tal, S Zofar) **17** *Singilis fuscoflavus* (Felix & Muilwijk, 2009) (Oman bor., Prov. Batinah) **18** *Singilis plagiatus* (Reiche & Saulcy, 1855) (Libanon, O v. Saida) **19** *Singilis libani* Sahlberg, 1913 (Israel, Golan Mach).

##### Variation.

In the holotype, last abdominal sternum with 6 setae (3 on each side), irregularly placed. One of the paratypes has 4 setae (two on each side) and one has 5 setae: 3 on one side and 2 on the other.

Aedeagus – Fig. 38. Aedeagal median lobe straight euventrally for much of length between basal bulb and slightly downturned, elongate, narrow apex with tightly rounded tip. Internal sac without apparent spicules.

##### Distribution.

UAE, Oman (country record).

#### Singilis fuscipennis

Schaum, 1857

http://species-id.net/wiki/Singilis_fuscipennis

Singilis fuscipennis Schaum 1857: 258

##### Material examined.

GREECE: Attica, Dr. Krüper (1♂, NHMW); Graecia, Parnass, Collect. Hauser (2♂♂, NHMW); Dedeagač, Graecia sept. (1♂, NMPC). SYRIA: Al Lathqiyan env., Slenfe env., 1500m, 30–31.V.1998 Josef Mertlik leg. (1♂, DWWC); Syria (1♀, NMPC). TURKEY: Anatolia m., Toros Daglari 900m, 25 km NW Erdemli, 6.VI.1991, S. Kadlec leg. (1♂, NHMW); Bogaz Roy, 12.VII.1989, H. Schmid leg. (1♂, NHMW); Asia Minor, Akschehir (1♂, NHMW); vill. Icel 24–26.V.1995, Erdemli env., 8 km NW of Arslanli, Josef Mertlik leg. . (1♂, DWWC); Asia min., 1.V.1969, Burdur, Antalya, Wewalka leg. (1♂, NMPC); prov. Adana, 20 km NW of Erdemli, 13–15.VI.1992, V. Bíša & Z. Koščál leg. (1♂, DWWC).

##### Diagnosis.

This species is most similar to *Singilis libani*, sharing a body form and coloration pattern, but is smaller and can be diagnosed easily by pronotum, i.e., *Singilis libani* has strongly transverse and very densely punctate pronotum. The aedeagal median lobe apex is broader and shorter than in *Singilis libani*.

##### Redescription.

Length 5.1–5.9 mm. Head and pronotum brown; elytra piceous to black, with paler lateral margins and a poorly defined triangular area in basal ¼, sometimes extended along suture (Fig. 23).

Head very deeply and densely punctate, strongly microsculptured; punctures sometimes confluent near eyes, separated by a diameter on the vertex. Clypeus impunctate. Eyes large and bulging, with a few very short setae at posterior margin. Second supraocular seta located just anterad the posterior margin of eye. Temples short and smooth. Scape with a very long seta at 2/3 of its length and a few short thin setae; pedicel with a band of apical setae; antennomere III with two bands of setae (at mid-length and at apex). Antennae pubescent from mid-length of antennomere IV.

Pronotum 1.23–1.27 times as wide as head, 1.45–1.5 times as wide as long, widest just behind marginal setae. Anterior margin straight, anterior angles slightly prominent, sides very broadly and evenly rounded, slightly to conspicuously sinuate towards posterior angles, which are acute. Punctation coarse, irregular and deep, somewhat sparser towards furrow, rugose and confluent towards lateral margins. Lateral explanate margin rapidly widened from the apex, broad and flat at base. Posterior pore right in front of angle. Basal grooves shallow, rugose, confluently punctate. Pronotal base extended in a rounded median lobe. Furrow variable. Microsculpture strong, slightly transverse.

Intervals 1, 3, 5 and 7 with subtle, irregular setiferous punctures bearing minute, barely visible setae. Interval 7 narrow and strongly convex. All intervals convex near base and conspicuously convex elsewhere. Striae deep from base to apex, slightly punctate. Microsculpture strong, polygonal. Apices slightly sinuate.

Legs brownish yellow. Tarsomere V with 3 pairs of ventral setae. Mes- and metepisterna slightly punctate. Claws with 5 teeth. Venter brown, abdominal sterna blackish. All abdominal sterna with short pubescence, no longer than metatarsomere IV.

Aedeagus – Fig. 39. Aedeagal median lobe stout, eudorsal surface slightly curved, apex broad, expanded slightly near broadly rounded tip.

**Figures 20–23:**
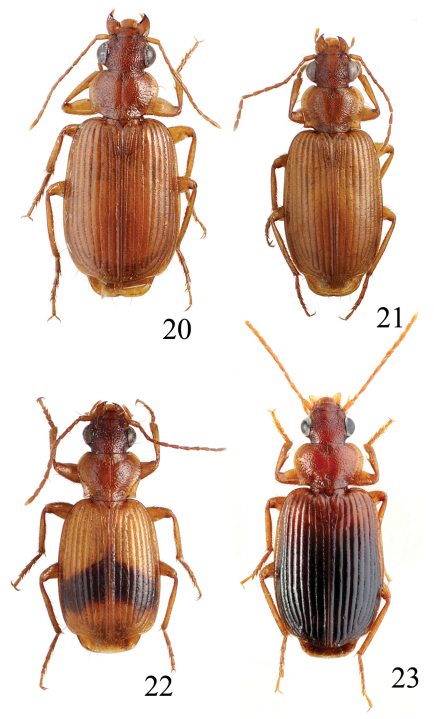
. Habitus of *Singilis*: **20** *Singilis kryzhanovskii* sp. n., Holotype **21** *Singilis saeedi* sp. n., Holotype **22** *Singilis loeffleri* Jedlička, 1963b, Holotype **23** *Singilis fuscipennis* Schaum, 1857 (Turkey).

##### Variation.

Varies in body size, in the extent of pale area at elytral base (may be reduced), and depth and length of the pronotal furrow.

##### Comments.

Type locality: Athens, Greece (Schaum 1857: 134).

##### Distribution.

Bulgaria, Greece, Turkey, Syria.

#### Singilis libani

J.R. Sahlberg, 1913 stat. n.

http://species-id.net/wiki/Singilis_libani

Singilis fuscipennis  var. *libani*Sahlberg 1913: 37.
Phloeozetus fuscipennis libani : Kabak 2003: 438.

##### Material examined.

ISRAEL: Palestina, Gebatha, 2.IX.1927, collectto Paganetti (1 ex. NHMW); Israel, Golan Mach, Pappelrinde 16.7.1985 (1♂, NHMW); Galil, 8.6.1999, Yoqne'am, I. Trojan (1♂, DWWC). SYRIA: Syria occ., distr. Tartus, Safita env., 400 m a.s.l., 30 km SE of Tartus, 1.V.2000, steppe, S. Benedikt leg. (1♂, DWWC).

##### Diagnosis.

This species is most similar to *Singilis fuscipennis*, with diagnostic differences listed under that species.

##### Redescription.

Length 6.8–7.0 mm. Head, pronotum, and legs brown, elytra piceous to black, sometimes slightly paler around scutellum. Venter brown, with darker abdomen (Fig. 19).

Head deeply microsculptured, very deeply and densely punctate, less or no more than one diameter distance from each other. Clypeus punctate in posterior half. Eyes big and prominent, with no short setae at posterior margin. Second supraocular seta located just anterad the posterior margin of eye. Temples short and smooth. Scape with a very long seta at 2/3 of its length and a few short thin setae towards the apex; pedicel with numerous setae in apical half; antennomere III pubescent towards apex. Antennae pubescent almost from the base of antennomere IV.

Pronotum 1.30–1.37 times as wide as head, 1.57 times as wide as long, widest just behind marginal setae. Anterior margin emarginate, anterior angles slightly prominent, sides very broadly rounded, not sinuate at posterior angles, which are rectangular and protrude as minute denticle. Punctation very coarse, as dense or denser than on head. Lateral explanate margin rapidly widened from the apex, broad and flat at base. Posterior pore right in front of angle. Basal grooves shallow, rugose, confluently punctate. Pronotal base extended in a rounded median lobe. Furrow usually shallow, fine, and incomplete. Microsculpture same as on head.

Elytra piceous to black, sometimes with base narrowly paler. Intervals 1 and 7 with a single row, 3 and 5 with two rows of irregular setiferous pores. Setae minute, barely visible. Interval 7 convex and narrow in basal half. Microsculpture polygonal to almost isodiametric. Apices slightly sinuate. Striae deep, punctate. All intervals convex at base, slightly convex at apex; outer intervals convex throughout.

Legs brownish yellow. Tarsomere V with 4–5 pairs of ventral setae. Claws with 4 long teeth. Epimera slightly rugose. Mes- and metepisterna smooth. Venter brownish yellow, with 3–4 apical sterna darker. Abdomen with short pubescence.

Aedeagus – Fig. 40. Aedeagal median lobe moderately broad, median shaft straight on ventral surface, apical sixth of lobe subangulately downturned, tip slightly pointed.

##### Comments.

Originally described as a subspecies of *Singilis fuscipennis* Schaum, 1857 (Sahlberg, 1913: 37) based on a single specimen from Jabal al-Baruk in central Lebanon (“Baruk Mt., Libani, IV.1819, Syr.").

##### Distribution.

Israel, Lebanon, Syria.

#### Singilis plagiatus

(Reiche & Saulcy, 1855)

http://species-id.net/wiki/Singilis_plagiatus

Coptodera plagiata Reiche and Saulcy 1855: 578.
Phloeozetus plagiatus plagiatus : Kabak 2003: 438.

##### Material examined.

Type: ♂, Syrie (MNHN). JORDAN: Petra, Taybeh, 21.V.1994, W.G. Ullrich (1♀, DWWC). LEBANON: Appl, Beirut, 1878 (2♂, NHMW); Libanon, O v. Saida, 9–16.V.1963 Kasy & Vartian; “Phloeozetaeus apicalis Jedl. det. Ing. Jedlička" (1♂, NHMW).

##### Diagnosis.

Among the species consisting of beetles with strongly transverse and very densely punctate pronotum, easily diagnosable by bicoloured elytra, with apical half black.

##### Redescription.

Length 5.5–6.2 mm. Head, pronotum and ventral segments ferrugineous, apical half of elytra piceous (Fig. 18).

Head very coarsely and deeply punctate on the sides and towards base, more sparsely on the frons, and very distinctly microsculptured. Punctures near eyes often confluent. Clypeus smooth, with distinctly microsculpture. Eyes large and bulging, with no short setae at the posterior margin. Second supraocular seta located just anterad the posterior margin of eye. Temples very short, smooth. Scape with several setae besides the very long subapical one; pedicel with a band of apical setae; antennomere III in apical half with several setae besides the usual apical ones. Antennae pubescent from the basal fourth of antennomere IV.

Pronotum transverse, 1.17 times as wide as head, 1.4–1.48 times as wide as long, widest just behind marginal setae. Anterior margin straight, anterior angles effaced or faintly marked, sides broadly and evenly rounded, sinuate towards posterior angles, which are acute. Punctation coarse and irregular, punctures (especially on disc) generally smaller and sparser than on head, sometimes confluent (especially on the sides of disc). Lateral explanate margin uniform near apex, rapidly widened, wide and flat towards base. Posterior pore right in front of angle. Basal grooves shallow. Pronotal base extended in a rounded median lobe. Furrow variable. Microsculpture less distinct than on head.

Elytra shinier than head and pronotum. Each interval with 1–2 rows of minute, inconspicuous setae. Microsculpture more delicate than on head and pronotum. Apices weakly obliquely sinuate, rounded at suture. Striae slightly punctate, deep at base and at shoulders, shallower on disc. Interval 7 not much more convex and narrow than interval 6. Intervals slightly convex at base, flat at apex.

Legs brownish yellow. Protarsomere V with 3 pairs of ventral setae, metatarsomere V with 4 pairs. Claws with 4 teeth. All abdominal sterna pubescent, pubescence as long as on tarsi.

Aedeagus – Fig. 41. Aedeagal median lobe slightly arcuate to apex, apex long. Internal sac with microtrichial patches composed of extremely small spicules.

##### Variation.

Varies in body size, density and size of pronotal punctures, and length of furrow.

##### Comments.

Type locality per original description is Beirut (Reiche and Saulcy 1855: 578–579).

##### Distribution.

Jordan, Lebanon. (Iraq, Israel, and Saudi Arabia records need confirmation.)

#### Singilis filicornis

Peyerimhoff, 1907

http://species-id.net/wiki/Singilis_filicornis

Singilis (Phloeozetaeus) filicornis Peyerimhoff 1907: 8.
Phloeozetus filicicornis : Kabak 2003: 438; Lorenz 2005: 480.

##### Material examined.

Type: ♂, Sinai, ouadi Sa'al, 25.II.1902; Sinai, O. Saal (1♀, MNHN); ISRAEL: Negev 12 km NW Elat, Oberh. En Natafim, 17.II.1987, 600m, Schawaller & Schmalfuss leg. (1♀, SMNS); Negev, Meshar-Ebene, 14.II.1987, 300 m, Schawaller & Schmalfuss leg. (1♀, SMNS); Negev, 12 km NW Elat, Oberh. En Natafim, 17.II.1987, 600m, Schawaller & Schmalfuss leg. (1♀, DWWC).

##### Diagnosis.

This species is most similar to the allopatric *Singilis jedlickai*, new species. The two species can be diagnosed easily by microsculpture of pronotum, i.e., *Singilis jedlickai* has conspicuously microreticulate pronotum. The aedeagus also differs dramatically (Figs 42–43).

##### Redescription.

**Figures 24–27.**
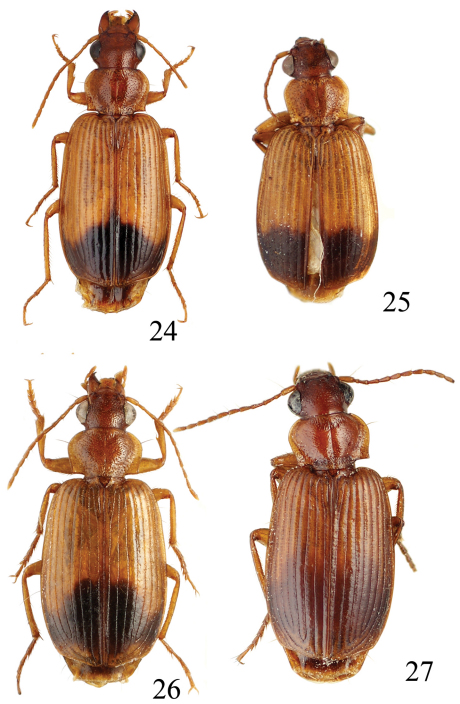
Habitus of *Singilis*: **24** *Singilis filicornis* Peyerimhoff, 1907 (Israel, Negev) **25** *Singilis jedlickai* sp. n., Holotype **26** *Singilis felixi* sp. n., Paratype (Oman bor., Prov. Batinah) **27** *Singilis persicus* (Jedlička, 1961a), Holotype.

Length 6.8–7.0 mm. Reddish brown, with apical third of elytra black (Fig. 24).

Head shiny, densely minutely punctate, punctures sometimes almost confluent in frontal depressions, separated by 1–2 diameters on the frons. Microsculpture scarcely visible. Clypeus with a few punctures near lateral margins. Eyes large and bulging, with 3–4 setae at posterior margin. Second supraocular seta located just anterad the posterior margin of eye. Temples smooth, 2.6 times as short as length of eye. Scape with a very long seta at 2/3 of its length and a few short thin setae towards the apex; pedicel with a band of apical setae; antennomere III with two bands of setae (at mid-length and at apex). Antennae pubescent from mid-length of antennomere IV.

Pronotum shiny, without microsculpture, 1.12–1.14 times as wide as head, 1.33–1.4 times as wide as long, widest just behind the marginal setae. Anterior margin straight, anterior angles effaced, sides evenly rounded, considerably to slightly sinuate towards posterior angles, which are acute and protrude as minute denticle. Punctation, especially on disc, sparse, irregular, somewhat sparser than on head, denser towards lateral margins, where may be confluent and rugose. Lateral explanate margin widened basad lateral setae, broad and flat at base. Posterior pore right in front of angle. Basal grooves small and shallow. Pronotal base extended in a rounded median lobe. Furrow fine.

Intervals 1, 3, 5 and 7 each from base to apex with an irregular row of sparse pores bearing extremely short, barely visible setae. At humerus, interval 7 flat or weakly convex and as wide as the adjacent intervals. Microsculpture delicate, polygonal. Apices slightly sinuate. Striae finely punctate. Intervals slightly convex at base and almost flat at apex.

Legs brownish yellow. Tarsomere V with 4 pairs of ventral setae. Propleuron, mes- and metepisterna smooth, shiny. Claws with 3 short teeth and one minute denticle at base. Venter uniformly testaceous. Abdominal sterna smooth, shiny, with sparse long pubescence.

Aedeagus – Fig. 42. Aedeagal median lobe arcuate, apex long; internal sac with small ventral microtrichial field composed of small spines.

##### Distribution.

Egypt, Israel. Iraq and Saudi Arabia records need confirmation.

#### Singilis jedlickai

sp. n.

urn:lsid:zoobank.org:act:4ED74ABF-B482-45CA-9653-A509EF687534

http://species-id.net/wiki/Singilis_jedlickai

##### Material examined.

Holotype: ♂, Afganistan, Kaboul // *apicalis* det. Ing. Jedlička (NMPC).

##### Diagnosis.

Resembles *Singilis filicornis* in body shape, elongate elytra, the elytral spot, and the slight pronotal punctation, but can be easily distinguished by presence of well-developed spicular fields, composed of large spines, on the male internal sac (Fig. 43).

##### Description.

Length 6.8 mm. Yellowish red-brown, apical third of elytra pitch-brown (Fig. 25).

Head densely finely punctate, slightly isodiametrically microsculptured; punctures confluent and rugose near eyes, separated by 1 to 3 diameters on the frons. Clypeus smooth. Eyes large and bulging, with 3–4 short setae at the posterior margin. Second supraocular seta located just anterad the posterior margin of eye. Temples smooth, 2.6 times as short as eye length. Scape with a very long seta at 2/3 of its length and a few short thin setae towards the apex; pedicel with the usual band of apical setae; antennomere III with two bands of setae, at the middle and apex. Antennae pubescent from mid-length of antennomere IV.

Pronotum 1.13 times as wide as head, 1.28 times as wide as long, widest just behind the marginal setae. Anterior margin slightly concave, sides behind marginal setae straight, sinuate towards posterior angles, which are rectangular and protrude as minute denticle. Punctation sparse, irregular, punctures (especially on disc) sparser than on head, transversely rugose and confluent along lateral margins. Lateral explanate margin widened from the marginal setae, broad and flat at base. Posterior pore right in front of angle. Basal grooves small and shallow. Pronotal base extended in a rounded median lobe. Furrow thin. Microsculpture faint, isodiametric.

Elytra elongate, in middle 1.46 times as long as wide. Intervals 1, 3, 5 and 7 with conspicuous pores bearing minute, barely visible setae. Microsculpture polygonal. Apices slightly sinuate, rounded at suture. Striae deep and minutely punctate. Intervals 3–7 convex at humerus, gradually flattened towards apices; other intervals slightly convex at base and almost flat at apex.

Legs pale brownish yellow. Tarsomere V with 4 pairs of ventral setae. Claws with three long and one small basal teeth. Venter entirely brownish yellow. Propleuron faintly rugose, episterna smooth. All abdominal sterna with short pubescence.

Aedeagus – Fig. 43. Aedeagal median lobe broad, ventral surface almost straight to apex, apical fourth of lobe slightly downturned. Apex of median lobe long and expanded slightly near rounded tip. Internal sac with huge fields of numerous long and large spines.

##### Name derivation.

Named after the eminent Czech coleopterist Arnost Jedlička.

##### Distribution.

Known from a single male from Kaboul, Afghanistan.

#### Singilis felixi sp. n.

urn:lsid:zoobank.org:act:155D08B4-9888-4DCA-8D2A-E7991EA0A2C6

http://species-id.net/wiki/Singilis felixi

##### Material examined.

OMAN: Oman bor., Prov. Batinah, Al-Jabal al-Ahdar mts., SE Rustaq, W Awabi, 430 m, Wadi Bani Awi, 23°20'0.13"N, 57°29'23.5"E, L. fang + L. fallen, 29–30.XII.2009 leg. Lehmann, Bittner & Stadie (4♂♂ 1♀, AAC, APC). UAE: HOLOTYPE: ♂, UAE, Bithnah, 25.10N 56.14E, 31.XII.2005–29.I.2006, light trap, A. v. Harten (UAEIC); PARATYPES: UAE, Hatta, 24.49N, 56.07E, 8–24.IV.2006, light trap, A. v. Harten (1♂, RFC); UAE, Wadi Safad, 25.13N 56.19E, 20.XII.2005–2.I.2006, light trap, A. v. Harten (2♂♂, RMNH, RFC); UAE, Wadi Safad, 25.13N,  56.19E, 21.02–4.III.2006, light trap, A. v. Harten (1♀, RFC).

##### Diagnosis.

Extremely similar to *Singilis persicus*, can be diagnosed based on the length of hairs on abdominal sterna. In *Singilis felixi* the hairs are long, the same or at least 2/3 of length of the apical setae (shorter in *Singilis persicus*); odd intervals of elytra in *Singilis felixi* with easily visible short setae along striae (tiny and difficult to see in *Singilis persicus*).

##### Description.

Length 7.0–8.6 mm. Pale yellowish red-brown, elytra with large piceous preapical spot (Fig. 26).

Head deeply irregularly punctate, microsculptured, punctures sometimes almost confluent in frontal depressions, separated by over three diameters on the frons. Clypeus with few punctures near lateral margins. Eyes large and bulging, with a few short setae at posterior margin. Second supraocular seta located just anterad the posterior margin of eye. Temples short and smooth. Scape with a very long seta at 2/3 of its length and a few short thin setae towards apex; pedicel with the usual band of apical setae; antennomere III with two bands of setae at mid-length and at apex. Antennae pubescent from mid-length of antennomere IV.

Pronotum shinier than head and elytra, 1.16–1.18 times as wide as head, 1.39–1.42 times as wide as long, widest just behind marginal setae. Anterior margin straight, anterior angles effaced, sides very broadly and evenly rounded, slightly sinuate towards posterior angles, which are acute and protrude as minute denticle. Punctation coarse, irregular, deep, somewhat sparser than on head, especially on disc; rugose and confluent at apical and basal margins. Lateral explanate margin rapidly widened from the apex, broad and flat at base. Posterior pore right in front of angle. Basal grooves shallow, rugose, confluently punctate. Pronotal base extended in a rounded median lobe. Furrow deep and complete. Microsculpture faint.

Elytra pale yellowish red-brown, with red brown apices and piceous preapical spot reaching lateral margins (may be reduced to five inner intervals). Intervals 1, 3, 5 and 7 each with minute setae and a row of pores from base to apex. Interval 7 flat, as wide as adjacent intervals. Microsculpture deep, irregular, polygonal, same as on head. Apices slightly sinuate. Striae slightly punctate, shallower on disc and at apex. Intervals slightly convex near base, flat at apex.

Legs brownish yellow. Tarsomere V with 3 pairs of ventral setae. Mes- and metepisterna slightly punctate. Claws with 5 teeth. Venter entirely light brownish yellow. All abdominal sterna with pubescence more than twice as long as protarsomere IV.

Aedeagus (Fig. 44), internal sac without apparent spines.

##### Variation.

Varies in body size; elytral spot sometimes reduced. Pronotal basal angles usually acute and prominent but sometimes rectangular and less prominent.

##### Name derivation.

Named after Ron Felix, my friend and collaborator who discovered this species.

##### Distribution.

Oman (country record), UAE.

#### Singilis persicus

(Jedlička, 1961a)

http://species-id.net/wiki/Singilis_persicus

Phloeozetaeus persicus  Jedlička, 1961a: 3.
Phloeozetus persicus : Kabak 2003: 438.

##### Material examined.

HOLOTYPE: ♀, S.O. Iran, Djiroft, Anbar-Abad, 1–18.V.1956, W. Richter leg. (SMNS).

##### Diagnosis.

This species is most similar to *Singilis felixi*, with diagnostic differences listed under that species.

##### Redescription.

Length 7.0 mm. Reddish yellow, with apical third of elytra dark (Fig. 27).

Head microsculptured and rather coarsely punctate. Clypeus punctate in basal half. Eyes with a few small setae at the posterior margin. Second supraocular seta located just anterad the posterior margin of eye. Temples short and smooth. Scape with a very long seta at 2/3 of its length and a few short thin setae towards the apex; pedicel with a band of apical setae; antennomere III with a few setae on apical half. Antennae pubescent from the basal fourth of antennomere IV.

Pronotum brownish yellow, shinier than head and elytra, 1.2 times as wide as head, 1.46 times as wide as long, widest behind the marginal setae. Anterior margin straight, anterior angles effaced, lateral margin very broadly and evenly rounded from anterior angle to marginal setae, almost straight behind that point, slightly sinuate towards posterior angles, which are acute and protrude as minute denticle. Punctation sparse, especially on disc, rugose and confluent at apical and basal margins. Lateral explanate margin widened behind marginal setae, broad and flat at base. Posterior pore right in front of angle. Basal grooves shallow, rugose, confluently punctate. Pronotal base extended in a rounded median lobe. Furrow deep and complete. Microsculpture faint.

Elytra: striae fine, finely punctate. Intervals 1, 3, 5 and 7 with extremely tiny and short setae and row of small pores from base to apex, very difficult to see. Interval 7 at shoulder slightly convex and narrower than the adjacent ones. Microsculpture deep, irregular, polygonal. Intervals slightly convex at base and almost flat at apex.

Legs brownish yellow. Tarsomere V with 3 pairs of ventral setae. Episterna of meso- and metathorax slightly punctate. Claws with 3–4 obtuse teeth. Venter entirely light brownish yellow. All abdominal sterna with pubescence short, no longer than protarsomere IV.

Male unknown.

##### Distribution.

Iran.

#### Singilis kryzhanovskii sp. n.

urn:lsid:zoobank.org:act:A2F324F9-77EC-4146-8C64-DB9897B38A34

http://species-id.net/wiki/Singilis_kryzhanovskii

##### Material examined.

Holotype: ♂, Iran, Khorasan prov., Gonobad area, 10 km SW Khezri, 1800m, 22.V.2009 Kolesnichenko K. leg.; Paratype: ♂, Kopetdag, 12 km SW Kizyl-Arvat, light trap, 5.VII.1953 Kryzhanovskii leg. (ZIN).

##### Diagnosis.

This new species shares with *Singilis klimenkoi* and *Singilis saeedi* the overall appearance and uniformly brownish yellow body coloration. It differs from *Singilis klimenkoi* by its larger size and by the short and thin setae on elytral intervals. From *Singilis saeedi* it is differentiated by the presence of weak setiferous pores along only odd elytral intervals, i.e. all elytral intervals of *Singilis saeedi* with pores along striae. The aedeagus also differs dramatically.

##### Description.

Length 6.3–6.5 mm. Uniformly yellowish red-brown (Fig. 20).

Head very coarsely and deeply irregularly punctate, with distinct isodiametric microsculpture, punctures confluent near eyes, separated by over three diameters on the frons. Clypeus smooth, with a few small punctures near base. Eyes very large and bulging. Second supraocular seta just before the level of the posterior margin of the eye. Temples very short, smooth. Scape smooth, with one very long and 3–4 short subapical setae; pedicel with the usual band of apical setae; antennomere III with two bands of apical setae. Antennae pubescent from the basal third of antennomere IV.

Pronotum 1.16 times as wide as head, 1.36–1.42 times as wide as long, widest just behind marginal setae. Anterior margin straight, anterior angles effaced, sides broadly and evenly rounded, slightly sinuate towards posterior angles, which are rectangular and protrude as minute denticle. Punctation irregular, punctures (especially on disc) shallower and sparser than on head, confluent at basal grooves. Lateral explanate margin even from apex to marginal setae, then rapidly widened and flat. Posterior pore right in front of angle. Basal grooves shallow. Pronotal base extended in a rounded median lobe. Furrow deep. Microsculpture as on head.

Elytra subparallel, 1.4 times as long as wide. Intervals 1, 3, 5 and 7 with small pores bearing minute inconspicuous setae. Microsculpture as on pronotum. Apical edge straight, rounded at suture. Striae deep, slightly punctate. Intervals 4–8 convex in basal half, gradually flattened towards apex; other intervals slightly convex at base and flat at apex.

Legs pale brownish yellow. Tarsomere V with 5 pairs of ventral setae. Claws with 5 teeth. Episterna all smooth. Abdominal sterna with long pubescence.

Aedeagus – Fig. 45. Aedeagal median lobe moderately broad, ventral surface straight at midlength, apical fourth of lobe slightly downturned. Apex of median lobe long and expanded slightly near rounded tip. Internal sac with numerous long and large spines.

##### Variation.

In the holotype, elytral intervals are more convex and striae more crenulate than in the paratype, whose striae are almost smooth. However the aedeagal configuration is identical, and so the two specimens are considered conspecific.

##### Name derivation.

Named after the eminent Russian coleopterist Oleg Kryzhanovsky.

##### Distribution.

Iran, Turkmenistan.

#### Singilis saeedi sp. n.

urn:lsid:zoobank.org:act:E12D7EB6-A5D0-4360-BBB8-CEEE4B3387BF

http://species-id.net/wiki/Singilis_saeedi

##### Material examined.

Holotype: ♂, Iran, Fars, 20 km W Estahban, 2400m, 27–30.V.2008 Anichtchenko A. leg. (ZIN). Paratypes: same locality and date (2♂♂ 4♀♀, ZIN, AAC).

##### Diagnosis.

This new species shares with *Singilis klimenkoi* and *Singilis kryzhanovskii* the overall appearance and body coloration, but differs from *Singilis klimenkoi* by the short and thin setae on elytral intervals. From the other it is differentiated by smaller body size, and presence of weak setiferous pores along all elytral striae, i.e. *Singilis kryzhanovskii* has only on odd elytral intervals.

##### Description.

Length 5.7–6.7 mm. Uniformly yellowish red-brown (Fig. 21).

Head very coarsely and deeply punctate on sides and towards the base, more sparsely on the frons; punctures near eyes often confluent. Head with very distinct microsculpture. Clypeus smooth, with distinct microsculpture. Eyes large and bulging, with no short setae at the posterior margin. Second supraocular seta located just anterad the posterior margin of eye. Temples very short, smooth. Scape with several setae besides the very long subapical one; pedicel with the usual band of apical setae; antennomere III in apical half with several setae besides the usual apical ones. Antennae pubescent from the basal fourth of antennomere IV.

Pronotum 1.17 times as wide as head, 1.4–1.48 times as wide as long, widest just behind marginal setae. Anterior margin straight, anterior angles effaced to faintly marked, sides broadly and evenly rounded, sinuate towards posterior angles, which are acute. Punctation coarse, irregular, punctures (especially on disc) generally smaller and sparser than on head, sometimes confluent (especially on the sides of disc). Lateral explanate margin uniform near apex, rapidly widened, wide and flat towards base. Posterior pore right in front of angle. Basal grooves shallow. Pronotal base extended in a rounded median lobe. Furrow variable. Microsculpture less distinct than on head.

Elytra shinier than head and pronotum. Each interval with 1–2 rows of minute, inconspicuous setae. Microsculpture more delicate than on head and pronotum. Apices weakly obliquely sinuate, rounded at suture. Striae slightly punctate, deep at base and at shoulders, shallower on disc. Interval 7 not much more convex and narrow than interval 6. Intervals slightly convex at base, flat at apex.

Legs brownish yellow. Protarsomere V with 3 pairs of ventral setae, metatarsomere V with 4 pairs. Claws with 4 teeth. Abdominal pubescence as long as tarsal, with no long setae.

Aedeagus – Fig. 46. Aedeagal median lobe stout, eudorsal surface slightly curved, apical third of lobe downturned, apex broad. Internal sac with three long and large spines and one field of small spicules.

##### Variation.

Varies in body size, density and size of pronotal punctures, and length of furrow.

##### Name derivation.

Named after my friend Saeed Mobarra.

##### Distribution.

Iran.

#### Singilis hirtipennis

Pic, 1901

http://species-id.net/wiki/Singilis_hirtipennis

Singilis (Phloeozeteus) hirtipennis Pic 1901: 89
Phloeozetus hirtipennis : Kabak 2003: 438.

##### Type.

Syrie, Monts Amanus, Delagrande (in coll. Pic, MNHN). Not examined.

##### Comments.

I failed to locate type specimens in MNHN, including the Pic collection. I have examined one female determined as *Phloeozetus plagiata* (“ex Musaeo Chaudoir // Museum Paris 1952, coll. R. Oberthür // *plagiata* Reiche, Syrie, Kindermann") and reasonably matching the original description of *Singilis hirtipennis*; I tentatively determined it as *Singilis loeffleri*; if true, *Singilis loeffleri* should be considered a junior synonym of *Singilis hirtipennis*, but resolving this case would require examining the type of the latter.

#### Singilis loeffleri

Jedlička, 1963b

http://species-id.net/wiki/Singilis_loeffleri

Singilis loeffleri Jedlička 1963b: 176

##### Material examined.

Holotype: ♂, Persien, Löffler (=Iran, Kuh-rang) (NMPC); Iran, Kerman, Sirjan, 8 km N Balvard, 11–12.V.2007, Anichtchenko A. leg. (1♂, CAA).

##### Diagnosis.

In *Singilis loeffleri*, the apex of the aedeagus is short and robust (long and slender in *Singilis turcicus*, Fig. 36), and the entire propleuron smooth (subrugose near coxae in *Singilis turcicus*).

##### Redescription.

Length 5.4 mm. red-brown with postmedian black band on elytra (Fig. 22).

Head smooth, microsculptured, deeply irregularly punctate, punctures sometimes almost confluent near eyes, separated by 3 to 6 diameters on the front and by their diameter towards head base. Clypeus impunctate. Eyes large and bulging, with a few short setae at posterior margin. Second supraocular seta located just anterad the posterior margin of eye. Temples short and smooth. Scape with a very long seta at 2/3 of its length and a few thin setae towards the apex; pedicel irregularly setose throughout; antennomere III with numerous setae in apical 2/3. Antennae pubescent from the basal fourth of antennomere IV.

Pronotum red-brown, smooth, shinier than head, 1.21 times as wide as head, 1.34 times as wide as long, widest right behind marginal setae. Anterior margin straight, anterior angles slightly prominent, sides very broadly and regularly rounded, slightly to moderately sinuate towards posterior angles, which are rectangular to acute. Punctation irregular, sparser and shallower than on the head, sparse on the disc and denser towards base. Lateral explanate margin rapidly widened from apex, broad and elevated at base. Posterior pore right in front of angle. Basal grooves punctate. Pronotal base extended in a rounded median lobe. Furrow short and shallow. Microsculpture weak, slightly transversely polygonal.

Elytra red-brown, with piceous to black postmedian band reaching lateral margins. Intervals 1, 3, 5, 7 and 8 setose, with a single irregular row of pores all along. All intervals setose at base, convex on basal third and slightly convex beyond that. Interval 7 convex from base to the middle. Microsculpture deep, irregular, polygonal. Apical margin slightly sinuate. Striae deep, punctate, shallower towards the apices.

Legs brownish yellow. Tarsomere V with 3 pairs of ventral setae. Mes- and metepisterna smooth. Claws with 4 teeth. Venter entirely light brownish yellow. Abdominal sterna pubescent throughout, pubescence as long as protarsomere II.

Aedeagus – Fig. 47. Aedeagal median lobe moderately broad dorsoventrally, ventral margin straight nearly to apex; apex with evenly rounded tip that is not downturned.

##### Variation.

Elytral band may be reduced to a spot not reaching beyond interval 4. Pronotal and elytral punctures vary in size.

##### Comments.

Jedlička (1963b: 177) erroneously referred to the holotype as female. *Singilis loeffleri* is probably conspecific with *Singilis hirtipennis*, as discussed above.

##### Distribution.

Iran.

#### Singilis timidus sp. n.

urn:lsid:zoobank.org:act:5287A9A3-8591-476B-AB4C-1E3F1267A607

http://species-id.net/wiki/Singilis_timidus

##### Material examined.

Holotype: ♂, small green rectangle; white label in Russian: “coll. Khristofa" [‘Christoph Collection']; white label in Russian: “na osnovanii tetradi etiketka: Schahrud, Persia 1870–1873" [‘Location per log: Schahrud, Persia 1870–1873'] (ZIN); Paratype: ♀, Iran, Lorestan, 1–2.V.2007, 20 km N Pol-e-Dokhtar, Baba Zeyd, near Muruni, Anichtchenko A. leg. (AAC).

##### Diagnosis.

This new species can be confused with specimens of sympatric *Singilis mesopotamicus* with reduced elytral pattern. The two species can be diagnosed by aedeagal structure.

##### Description.

Length 6.1–6.2 mm. Yellowish red-brown, with legs slightly paler and a dark postmedian elytral spot reaching interval 4 (Fig. 7).

Head coarsely and deeply irregularly punctate, dull, very distinctly microsculptured. Punctures in the frons sometimes separated by over twice the diameter. Clypeus with some small irregular punctures. Eyes very large and bulging, with no small setae at posterior margin. Second supraocular seta located just anterad the posterior margin of eye. Temples short, smooth. Scape with several rather long setae besides the very long subapical one; pedicel with the usual band of apical setae; antennomere III with two bands of apical setae. Antennae pubescent from the basal third of antennomere IV.

Pronotum brownish yellow, 1.24 times as wide as head, 1.4 times as wide as long, widest just behind marginal setae. Anterior margin straight, anterior angles slightly marked, sides broadly and evenly rounded, more or less sinuate towards posterior angles, which are rectangular and protrude as minute denticle. Punctation coarse, irregular and deep, as dense as on head, somewhat sparser on disc and sometimes confluent, especially at the sides of disc. Lateral explanate margin rather wide at apex, rapidly widened basally, broad and flat towards base. Posterior pore right in front of angle. Basal grooves shallow. Pronotal base extended in a rounded median lobe. Furrow variable. Microsculpture less distinct than on head.

Elytra shinier than head and pronotum, 1.4 times as long as wide. Intervals 1, 3, 5 and 7 each with a row of setiferous pores. Pores of intervals 5 and 7 deep, with rather long setae. Intervals 2 and 4 with several minute setae at base. Microsculpture rather isodiametric but irregular, more delicate than on head and pronotum. Apices weakly obliquely sinuate, rounded at suture. Striae deep and crenulate. Interval 7 narrow and convex in basal half, then gradually flattened; other intervals convex at base, almost flat at apex.

Legs pale brownish yellow. Tarsomere V with 4 pairs of ventral setae. Claws with 4 teeth. Venter uniformly testaceous. All abdominal sterna with long pubescence. All episterna smooth.

Aedeagus – Fig. 48. Median lobe apex elongate, slightly downturned at narrowly rounded apex. Internal sac with well-developed spicular fields.

##### Variation.

Posterior pronotal angles almost rectangular in the holotype, more prominent and acute in the paratype.

##### Name derivation.

The name (Latin, adjective: timid) refers to the cryptic lifestyle.

##### Distribution.

Iran.

## Key to species of Singilis

**Table:** 

1	Body elongate (Figs 1–3), elytra 1.58–1.63 times as long as wide in the middle. Small size species, 4–5 mm.	2
–	Body wide (Figs 4–27), elytra 1.33–1.47 times as long as combined width in the middle. Size variable	4
2	Bicolored	3
–	Body completely black. (“Siberia", unspecified)	*Singilis anthracinus* (Solsky, 1874)
3	Pronotal sides sinuate in front of base, hind angles acute (Fig. 1). Legs all yellow. Endophallus without sclerotized denticles. (Afghanistan, Iran, Kyrgyzstan, Kazakhstan, Tajikistan, Turkmenistan, Uzbekistan)	*Singilis flavipes* (Solsky, 1874)
–	Pronotal sides less sinuate in front of base, hind angles rectangular. Femora and apical part of tibiae piceous to black (Figs 2–3). Endophallus with several sclerotized denticles. (S. Russia, Afghanistan, Iran, Iraq, Israel, Kyrgyzstan, Kazakhstan, Tajikistan, Turkmenistan, Uzbekistan)	*Singilis cingulatus* (Gebler, 1843)
4	Pronotum strongly transverse, very densely punctate, distance between punctures less than their diameter (Figs 17–19)	5
–	Pronotum less transverse or subquadrate, distance between punctures more than their diameter	7
5	Elytra uniformly brownish yellow or piceous	6
–	Elytra bicoloured, with apical half black (Fig. 18). Pronotal punctation strong, crenulate, same as on the head. (Lebanon, Syria)	*Singilis plagiatus* Reiche & Saulcy, 1855
6	Body colour uniformly yellow pale. Intervals of elytra flat. Small size species 4.8–5.4 mm. (Fig. 17). (Oman, UAE)	*Singilis fuscoflavus* (Felix & Muilwijk, 2009)
–	Body piceous to black, pronotum and sometimes elytral base narrowly paler (Fig. 19). Pronotum dull, deeply and densely punctate, punctures spaced by less than their diameter. (Palestine, Israel, Lebanon)	*Singilis libani* (Sahlberg, 1913)
7	All elytral intervals with conspicuous setiferous pores. Pubescence long	8
–	Setae short and thin, sometimes very short and inconspicuous	14
8	Intervals 1–7 with a single uninterrupted row of setiferous pores, 8^th^ with two rows	9
–	All intervals of elytra with 2–3 irregular and dense rows of big setiferous pores	12
9	Elytra elongate, flat, with black postmedian transverse band and red-brown apices. 6.0–6.5 mm. (Fig. 4). (Kazakhstan, Turkmenistan, Uzbekistan)	*Singilis amoenulus* (Semenov, 1889)
–	Elytra subovate, convex. Smaller, 4.6–5.1 mm.	10
10	Uniformly yellow brownish	11
–	Elytra with weak, diffuse, grey postmedian band, sometimes reduced to sutural spot, intervals 2, 4 and 6 without setiferous pores (Fig. 8). (Afghanistan, Iran)	*Singilis kabakovi* sp. n.
11	All elytral intervals with a single uninterrupted row of widely spaced setiferous pores. Pronotum transverse. Head and disc of pronotum very sparsely and superficially punctate (Fig. 10). (Iran)	*Singilis klimenkoi* sp. n.
–	Rows of setiferous punctures on intervals 3–5 widely interrupted. Setiferous pores on all intervals large. Pronotum cordate. Head smooth, pronotum almost smooth. (Fig. 9). (Iran)	*Singilis kolesnichenkoi* sp. n.
12	Propleura smooth and shiny	13
–	Propleura with wavy rugae. Size bigger, 6.8–8 mm. Elytra longer, basal red spot almost reaching the middle of elytra (Fig. 5). (Afghanistan, Kyrgyzstan, Kazakhstan, Tajikistan, Turkmenistan, Uzbekistan)	*Singilis solskyi* nom. n.
13	Propleura smooth and shiny. Body 6 mm. Elytra shorter and wider, basal third red-brown (Fig. 6). Microsculpture of elytra very strong, isodiametric, surface seems crenulate. Setiferous pores on elytra deep and small, very often situated on a par in the same interval. (Tajikistan)	*Singilis makarovi* sp. n.
–	Small, 5.1 mm. Basal half of elytra red. Elytra shiny, lightly microsculptured. Setiferous pores on intervals large, almost never situated on a par in the same interval. Head and pronotum sparsely punctate (Fig. 11). (Uzbekistan)	*Singilis timuri* sp. n.
14	Body and elytra uniformly yellow-brown	15
–	Body bicolored	16
15	All elytral intervals sparsely and irregularly punctate along striae (Fig. 21). Disc of pronotum sparsely punctate. Body and elytra uniformly yellow-brown. (Iran)	*Singilis saeedi* sp. n.
–	Only odd elytral intervals with irregular and weak setiferous pores all along striae. Setae very thin and short, barely visible (Fig. 20). (Iran, Turkmenistan)	*Singilis kryzhanovskii* sp. n.
16	Head and pronotum red-brown. Elytra red-brown with black band or spot, or with black posterior third of elytra	17
–	Head and elytra piceous to black. Pronotum and narrow diffuse band on the base of elytra paler (Fig. 23). Intervals of elytra convex, odd intervals with irregular punctures and tiny setae. (Greece, Turkey)	*Singilis fuscipennis* (Schaum, 1857)
17	Smaller, up to 5 mm.	18
–	Larger, at least 6–7 mm.	21
18	Pronotum strongly transverse. Elytra with black sutural spot extended to interval 4 or 5 (Fig. 16). Pronotal punctures spaced twice as wide as those on the head. (Israel, Yemen)	*Singilis discoidalis* (Mateu, 1986)
–	Pronotum subquadrate or slightly transverse	19
19	Pronotum subquadrate, sparsely irregularly punctate, punctures 2–6 diameter distance from each other, lateral sides sinuate before acutely prominent posterior angles. Intervals of elytra convex on the base, setiferous pores on odd intervals conspicuous. Transverse band variable (Fig. 22). (Iran) (see also comments in text about *Singilis hirtipennis* Pic, 1901)	*Singilis loeffleri* Jedlička, 1963b
–	Pronotum slightly transverse, densely punctate, punctures 1–2 diameters distance from each other, same as in head	20
20	Elytra short. Transverse black band of elytra narrow, often reduced to inner intervals or disappeared (Figs 12–13). Body size 4.3–5 mm (Armenia, Iran, Turkey)	*Singilis turcicus* (Jedlička, 1963a)
–	Elytra more elongate. Transverse black band of elytra broad, almost reaching apices of elytra. Odd intervals in apical part with 2 rows of setae, even intervals with 1 row of sparse setae (Figs 14–15). Body size bigger, 5.0–6.3 mm (Afghanistan, Iran, Iraq, Pakistan, Turkey)	*Singilis mesopotamicus* Pic, 1901
21	Pronotum densely punctate, same as on the head. Black apical spot of elytra not reaching apices	22
–	Pronotum sparsely punctate, shiny. Black apical spot of elytra reaching apices	23
22	Black elytral spot narrow, occupying zone from suture to 3 or 4 interval, not reaching apices of elytra (Fig. 7). (Iran)	*Singilis timidus* sp. n.
–	Transverse black band of elytra broad, almost reaching apices of elytra (Figs 14–15)	*Singilis mesopotamicus* Pic, 1901
23	Pronotum conspicuously microsculptured	24
–	Pronotum shiny, without microsculpture (Fig. 24). Endophallus without spines. (Israel, Egypt)	*Singilis filicornis* Peyerimhoff, 1907
24	Odd intervals of elytra with easily visible short setae along striae	25
–	Odd intervals of elytra with extremely tiny and short setae along striae. Hairs on abdominal sterna short, length no more than 1/3 of length of the apical setae. Pronotum more strongly transverse, sparsely and irregular punctate, less dense than on the head, disc almost without punctures, slightly rugose (Fig. 27). (Iran)	*Singilis persicus* (Jedlička, 1961a)
25	Hairs on abdominal sterna long, the same or at least 2/3 of length of the apical setae. Pronotum weakly transverse, regularly punctate. (Fig. 26). Endophallus without spines. (Oman, UAE)	*Singilis felixi* sp. n.
–	Hairs on abdominal sterna short, length no more than 1/3 of length of the apical setae. Apical third of elytra black (Fig. 25). Endophallus with numerous spines. (Afghanistan)	*Singilis jedlickai* sp. n.

**Figure 28.**
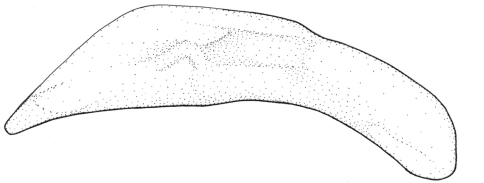
Aedeagus of *Singilis flavipes* (Solsky, 1874) (Kazakhstan: Karatau mt. rng., 40 km N Igilik vill.).

**Figure 29.**
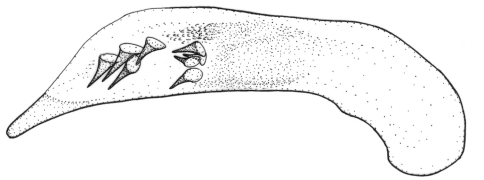
Aedeagus of *Singilis cingulatus* (Gebler, 1843) (Kazakhstan: Karatau mt. rng., 40 km N Igilik vill.).

**Figure 30.**
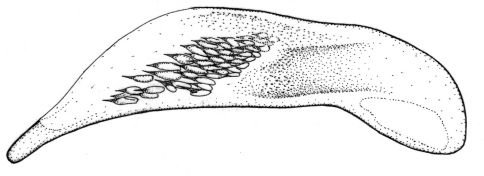
Aedeagus of *Singilis solskyi* nom. n. (Kazakhstan: Karatau mt. rng., 40 km N Igilik vill.).

**Figure 31.**
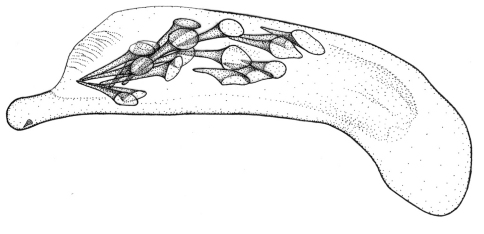
Aedeagus of *Singilis makarovi* sp. n., Holotype.

**Figure 32.**
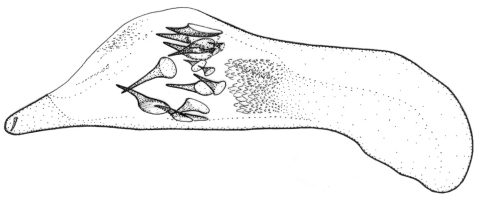
Aedeagus of *Singilis kabakovi* sp. n., Paratype (Iran: Khorasan, Torbat-e-Heydariyeh, 5 km S Zharf)

**Figure 33.**
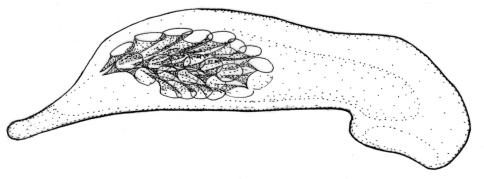
Aedeagus of *Singilis klimenkoi* sp. n., Holotype.

**Figure 34.**
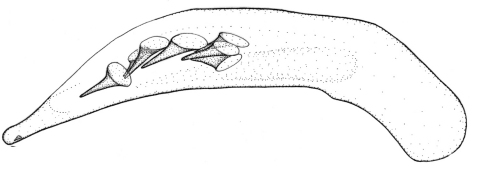
Aedeagus of *Singilis timuri* sp. n., Holotype.

**Figure 35.**
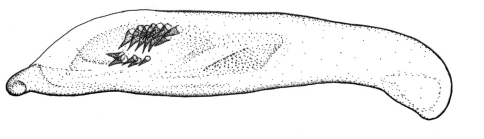
Aedeagus of S. *discoidalis* (Mateu, 1986) (Yemen: Lahj).

**Figure 36.**
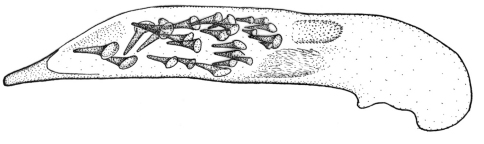
Aedeagus of *Singilis turcicus* (Jedlička, 1963a) (Armenia: Syunik prov., E Meghri, Artsvakar gorge).

**Figure 37.**
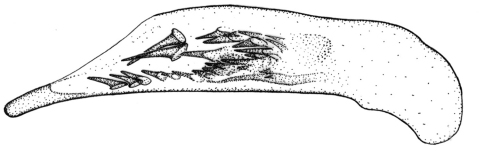
Aedeagus of *Singilis mesopotamicus* Pic, 1901 (Iran: Kerman, Qohrud mts., 10 km E Korin).

**Figure 38.**
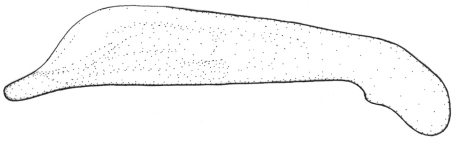
Aedeagus of *Singilis fuscoflavus* (Felix & Muilwijk, 2009) (Oman).

**Figure 39.**
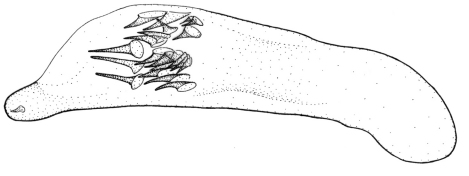
Aedeagus of *Singilis fuscipennis* Schaum, 1857 (Turkey: Bogaz Roy).

**Figure 40.**
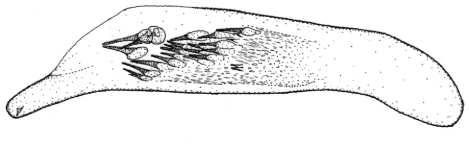
Aedeagus of *Singilis libani* Sahlberg, 1913 (Israel: Golan Mach).

**Figure 41.**
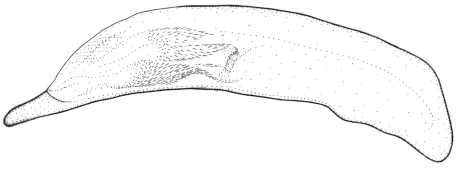
Aedeagus of *Singilis plagiatus* (Reiche & Saulcy, 1855) (Lebanon: O v. Saida).

**Figure 42.**
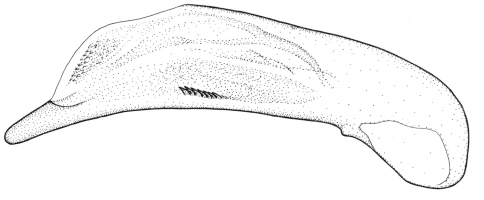
Aedeagus of *Singilis filicornis* Peyerimhoff, 1907, Holotype.

**Figure 43.**
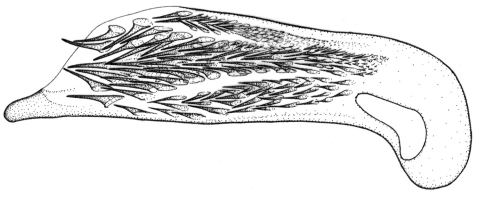
Aedeagus of *Singilis jedlickai* sp. n., Holotype.

**Figure 44.**
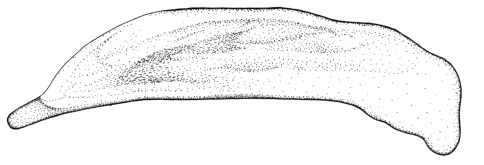
Aedeagus of *Singilis felixi* sp. n., Holotype.

**Figure 45.**
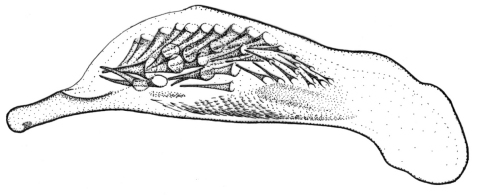
Aedeagus of *Singilis kryzhanovskii* sp. n., Holotype.

**Figure 46.**
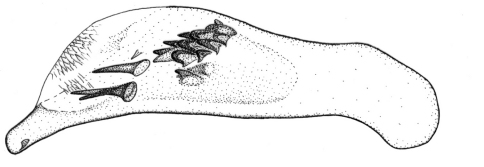
Aedeagus of *Singilis saeedi* sp. n., Holotype.

**Figure 47.**
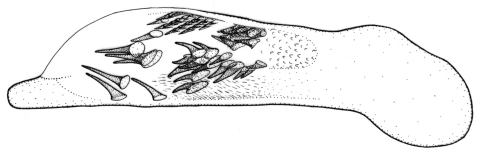
Aedeagus of *Singilis loeffleri* Jedlička, 1963b, Holotype.

**Figure 48.**
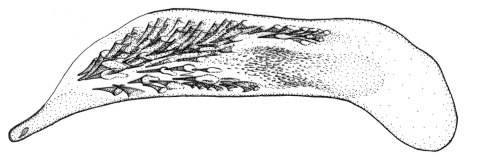
Aedeagus of *Singilis timidus* sp. n., Paratype (Iran: Lorestan).

## Supplementary Material

XML Treatment for Singilis
